# The Small Regulatory RNAs LhrC1–5 Contribute to the Response of *Listeria monocytogenes* to Heme Toxicity

**DOI:** 10.3389/fmicb.2018.00599

**Published:** 2018-03-27

**Authors:** Patrícia T. dos Santos, Pilar Menendez-Gil, Dharmesh Sabharwal, Jens-Henrik Christensen, Maja Z. Brunhede, Eva M. S. Lillebæk, Birgitte H. Kallipolitis

**Affiliations:** Department of Biochemistry and Molecular Biology, University of Southern Denmark, Odense, Denmark

**Keywords:** *Listeria monocytogenes*, heme toxicity, cell envelope stress, sRNA, two-component system, target mRNA

## Abstract

The LhrC family of small regulatory RNAs (sRNAs) is known to be induced when the foodborne pathogen *Listeria monocytogenes* is exposed to infection-relevant conditions, such as human blood. Here we demonstrate that excess heme, the core component of hemoglobin in blood, leads to a strong induction of the LhrC family members LhrC1–5. The heme-dependent activation of *lhrC1–5* relies on the response regulator LisR, which is known to play a role in virulence and stress tolerance. Importantly, our studies revealed that LhrC1–5 and LisR contribute to the adaptation of *L. monocytogenes* to excess heme. Regarding the regulatory function of the sRNAs, we demonstrate that LhrC1–5 act to down-regulate the expression of known LhrC target genes under heme-rich conditions: *oppA*, *tcsA,* and *lapB*, encoding surface exposed proteins with virulence functions. These genes were originally identified as targets for LhrC-mediated control under cell envelope stress conditions, suggesting a link between the response to heme toxicity and cell envelope stress in *L. monocytogenes*. We also investigated the role of LhrC1–5 in controlling the expression of genes involved in heme uptake and utilization: *lmo2186* and *lmo2185*, encoding the heme-binding proteins Hbp1 and Hbp2, respectively, and *lmo0484*, encoding a heme oxygenase-like protein. Using *in vitro* binding assays, we demonstrated that the LhrC family member LhrC4 interacts with mRNAs encoded from *lmo2186*, *lmo2185,* and *lmo0484*. For *lmo0484*, we furthermore show that LhrC4 uses a CU-rich loop for basepairing to the AG-rich Shine–Dalgarno region of the mRNA. The presence of a link between the response to heme toxicity and cell envelope stress was further underlined by the observation that LhrC1–5 down-regulate the expression of *lmo0484* in response to the cell wall-acting antibiotic cefuroxime. Collectively, this study suggests a role for the LisR-regulated sRNAs LhrC1–5 in a coordinated response to excess heme and cell envelope stress in *L. monocytogenes*.

## Introduction

*Listeria monocytogenes* is a Gram-positive, foodborne pathogen and the causative agent of listeriosis (Vazquez-Boland et al., 2001). To successfully establish an infection, this facultative intracellular pathogen must overcome several obstacles, such as low iron availability in the host (Chipperfield and Ratledge, 2000; Stojiljkovic and Perkins-Balding, 2002; Lungu et al., 2009). Upon infection, bacterial pathogens face a significant challenge in accessing iron, as it is mostly complexed to iron-binding proteins, such as hemoglobin, ferritin, lactoferrin, and transferrin (Hentze et al., 2004; Hammer and Skaar, 2011; Huang and Wilks, 2017). Bacterial pathogens are known to overcome this phenomenon, called nutritional immunity (Kochan, 1973; Wakeman and Skaar, 2012), through the activation of diverse mechanisms that allow iron acquisition from the iron-binding proteins in the host. Two-thirds of the total iron in the human body are sequestered in erythrocytes as heme bound to hemoglobin (Cassat and Skaar, 2013). In order to use heme as a source for iron, the pathogens first need to lyse erythrocytes, bind hemoglobin or other host heme proteins, and then extract and import the heme molecule for intracellular degradation to liberate free iron (Choby and Skaar, 2016). The iron acquisition system in *L. monocytogenes* has been the focus of diverse studies that have identified several specific iron transport and storage proteins (McLaughlin et al., 2011; Lechowicz and Krawczyk-Balska, 2015), such as Fhu, involved in the uptake of ferrichrome siderophores, and HupDGC, involved in the uptake of hemoglobin and hemin (i.e., the oxidized version of heme) (Jin et al., 2006; Xiao et al., 2011). Notably, HupDGC in *L. monocytogenes* is homologous to the well-studied iron-regulated surface determinant system (Isd) from *Staphylococcus aureus* (Skaar and Schneewind, 2004; Reniere et al., 2007). When the heme concentration in the environment is below 50 nM, heme acquisition in *L. monocytogenes* occurs with the participation of the heme-binding proteins 1 and 2, Hbp1 and Hbp2 (encoded by *lmo2186* and *lmo2185*, respectively), which are anchored in the cell wall by Sortase B (Xiao et al., 2011). While a role for Hbp1 is still unclear, Hbp2 is known to scavenge for heme and hemoglobin and facilitate the transport of heme through the cell wall (Xiao et al., 2011; Klebba et al., 2012; Malmirchegini et al., 2014). Heme can then cross the membrane through the HupDGC ABC transporter (Jin et al., 2006; Xiao et al., 2011). At higher heme concentrations, free heme molecules are predicted to diffuse through the porous structure of peptidoglycan. Then, they are bound by HupD anchored to the cytoplasmic membrane and transported into the cell (Klebba et al., 2012; Lechowicz and Krawczyk-Balska, 2015). Once inside the cell, heme may be used, for example, as a cofactor for several enzymes, such as catalases and peroxidases, as a respiratory cofactor for oxygen transport and storage, or as a catalyst of electron transfer (Chiabrando et al., 2014). Alternatively, heme can be broken down by heme oxygenases, like the characterized Isd-type heme-degradation enzyme Isd-LmHde and/or the IsdG homolog Lmo0484, to liberate free iron (Wu et al., 2005; Travaglini-Allocatelli, 2013; Duong et al., 2014). To maintain intracellular iron homeostasis, *L. monocytogenes* possesses an iron-binding protein, Fur (ferric uptake regulator), which negatively regulates several genes under iron-replete conditions, including the genes encoding Hbp1 and Hbp2 (Ledala et al., 2010). Fur boxes have also been identified upstream from other genes coding for proteins involved in the heme uptake and utilization system, such as HupDGC and IsdG/Lmo0484 (McLaughlin et al., 2012), suggesting that a tight regulatory connection between the iron and heme uptake/utilization systems is crucial for *L. monocytogenes*. Conversely, while heme can be an essential source of iron for *L. monocytogenes* upon infection, during certain pathological states, severe hemolysis may occur, resulting in high levels of free heme (up to 20 μM) (Arruda et al., 2004). As heme is a highly reactive lipophilic molecule, the cells must protect themselves against the potential damaging effects of heme under heme-rich conditions, such as in the bloodstream and blood-rich organs (McLaughlin et al., 2011; Choby and Skaar, 2016; Huang and Wilks, 2017). For *L. monocytogenes*, the mechanism by which this pathogen senses and responds to excess heme is yet to be characterized.

The LhrC family in *L. monocytogenes* consists of seven sibling sRNAs with regulatory roles under infection-relevant conditions (Thorsing et al., 2017). The family includes the highly homologous sRNAs LhrC1–5, as well as Rli22 and Rli33-1, which share lower homology. *rli22* and *lhrC1–5* are positively regulated by the two-component system (TCS) LisRK that responds to cell envelope stress, whereas the general stress sigma factor σ^B^ controls the expression of *rli33-1* (Sievers et al., 2014; Mollerup et al., 2016). The seven siblings are induced under various stress conditions and act to modulate the expression of specific target genes by an antisense mechanism. So far, three genes have been shown to be negatively regulated by the LhrCs at the post-transcriptional level: *lapB*, encoding a cell wall anchored virulence adhesin; *oppA*, encoding a substrate-binding protein of an oligopeptide transporter; and *tcsA*, encoding a CD4^+^ T cell-stimulating antigen (Sievers et al., 2014, 2015). For the mRNA targets characterized so far, the LhrC sRNAs act by basepairing to the Shine–Dalgarno (SD) region, leading to inhibition of translation initiation and/or decreased mRNA levels (Sievers et al., 2014, 2015; Mollerup et al., 2016). Strikingly, all seven sRNAs are highly induced when *L. monocytogenes* is exposed to human blood, suggesting an important regulatory role for the LhrC family in this host environment (Toledo-Arana et al., 2009). Yet, the specific component(s) in blood leading to induction of the LhrCs are presently unknown.

In this study, we aimed to investigate if the induction of the LhrC sRNAs by human blood could be linked to the increasing levels of heme in the bloodstream upon infection. Indeed, *L. monocytogenes* has the ability to secrete listeriolysin O (LLO) that facilitates the release of hemoglobin through erythrocyte lysis (Parrisius et al., 1986; Geoffroy et al., 1987; Foller et al., 2007). Similar to the LhrC family, *hly*, the gene encoding LLO, is also highly expressed in human blood, which supports the hypothesis that *L. monocytogenes* possibly encounters increasing levels of heme after host invasion (Toledo-Arana et al., 2009). Here, we demonstrate the LisR-dependent induction of LhrC family members, in particular LhrC1–5, by hemin, and show their capacity to regulate their known target genes under hemin stress conditions. In addition, we provide evidence that LhrC1–5 and LisR contribute to the adaptation of *L. monocytogenes* to excess hemin. Finally, we propose a role for LhrC1–5 in the post-transcriptional control of the heme uptake and utilization genes *lmo0484*, *lmo2185,* and *lmo2186* in response to cell envelope stress and excess hemin.

## Materials and Methods

### Bacterial Strains and Growth Conditions

The wild-type strain used in this study was *L. monocytogenes* serotype 1/2c strain LO28 (Vazquez-Boland et al., 1992). The isogenic mutant derivatives LO28Δ*lhrC1–5* and LO28Δ*lisR* were constructed in previous work (Kallipolitis et al., 2003; Sievers et al., 2014). The remaining isogenic mutant derivatives of this strain were constructed as previously described (Christiansen et al., 2004) by using the temperature-sensitive shuttle vector pAUL-A (Schaferkordt and Chakraborty, 1995). Primers used for in-frame deletions are listed in Supplementary Table S1. All strains used in this study are listed in Supplementary Table S2. *L. monocytogenes* was routinely grown at 37°C with aeration in brain heart infusion broth (BHI, Oxoid) unless stated otherwise. When appropriate, cultures were supplemented with kanamycin (50 μg/mL) or erythromycin (5 μg/mL). For induction of sRNA expression, cultures were supplemented with cefuroxime (4 μg/mL, corresponding to 9 μM) or various concentrations of hemin (Sigma). Hemin is the commercially available version of heme, which contains the oxidized Fe^3+^ ferric form instead of the reduced Fe^2+^ ferrous form. For experimental purposes, we will refer to hemin, while for the discussion we will refer to heme instead. Hemin was dissolved in 1.4 M NaOH and stock solutions were prepared fresh every time. In stress tolerance assays, overnight cultures were diluted to OD_600_ = 0.002 into BHI adjusted with various concentrations of hemin, or into BHI, when hemin was added to exponentially growing cells (OD_600_ = 0.2); growth was monitored until strains reached stationary phase. For cloning of plasmid vectors, *Escherichia coli* TOP10 cells (Invitrogen) were grown with aeration in Luria-Bertani broth supplemented with kanamycin (50 μg/mL) or erythromycin (150 μg/mL), when appropriate.

### Plasmid Constructions and β-Galactosidase Assays

To study the transcriptional activity of *lhrC1–5*, *rli22,* and *rli33-1*, we used the promoter-less *lacZ* transcriptional fusion vector pTCV-lac (Poyart and Trieu-Cuot, 1997) fused to the promoter regions of the seven sRNA-encoding genes constructed previously (Sievers et al., 2014; Mollerup et al., 2016). Post-transcriptional regulation of LhrC target genes was monitored using in-frame translational *lacZ* fusions of *lapB* constructed in previous work (Sievers et al., 2014), and of *lmo0484* constructed in the present study. Briefly, DNA fragments encoding a moderate promoter (Sievers et al., 2014) as well as a region spanning the SD region of *lmo0484* (-48 to +47, relative to translation start site) were ligated into pCK-lac (Nielsen et al., 2010). For the β-galactosidase assay, *L. monocytogenes* strains carrying the plasmids were grown overnight, diluted to OD_600_ = 0.02 into fresh BHI and grown to OD_600_ = 0.2 (for strains with translational fusions) or OD_600_ = 0.35 (for strains with transcriptional fusions). Cultures were then split and stressed with either 9 μM cefuroxime or 8 μM hemin for 1 and/or 2 h. Samples (1 mL) were withdrawn prior to stress and at the indicated time points after the subjected stress. β-galactosidase assays were conducted as previously described (Christiansen et al., 2004). As the applied stress conditions resulted in impaired growth relative to the non-stress control condition, a direct comparison between the stressed and non-stressed cultures was not possible. However, the growth of the wild-type and mutant strains were comparable under each of the conditions tested (i.e., control, cefuroxime or hemin stress, respectively). Therefore, the β-galactosidase activities of wild-type and mutant strains were analyzed for each of the conditions using two-tailed Student’s *t*-test (i.e., wild-type, stressed vs. mutant, stressed). Only differences with at least 95% confidence were reported as statistically significant.

### RNA Isolation and Purification

For primer extension and northern blot analysis, *L. monocytogenes* was grown to OD_600_ = 0.35. Cultures where then split, stressed with the indicated stressor concentration and samples were taken (10 mL) at the indicated time points. Cells were harvested by centrifugation at 11,000 rcf for 3 min at 4°C, and snap-frozen in liquid nitrogen. Cells were disrupted by the FastPrep instrument (Bio101, Thermo Scientific Corporation) and total RNA was extracted using TRI Reagent (Molecular Research Center, Inc.) as previously described (Nielsen et al., 2010). The integrity, concentration and purity of the RNA were confirmed by agarose gel electrophoresis, and NanoDrop 2000 or DeNovix DS-11 Fx+.

### Primer Extension

Primer extension experiments were performed as previously described (Christiansen et al., 2004). The ^32^P-labeled, single-stranded primers used for detection of *lmo0484* and *lmo2186* transcription start sites are listed in Supplementary Table S1.

### Northern Blotting

Total RNA (10 μg) was resolved on a 6% or 8% polyacrylamide-8 M urea gel as previously described (Nielsen et al., 2010); alternatively, 20 μg of total RNA was separated on a formaldehyde agarose gel for 3 h and 15 min prior to capillarity blotting on a Zeta-Probe membrane (Bio-Rad) (Sheehan et al., 1995). Membranes were hybridized with ^32^P-labeled DNA single-stranded probes listed in Supplementary Table S1. RNA bands were visualized using a Typhoon Trio or a Typhoon FLA9000 (GE Healthcare) and analyzed with IQTL 8.0 quantification software (GE Healthcare).

### Reverse Transcriptase-Quantitative Polymerase Chain Reaction (RT-qPCR)

Fifty μg of total RNA was DNase-treated according to the manufacturer (Roche), and further purified with phenol-chloroform extraction. Three μg of DNA-free RNA was used for cDNA synthesis using the Maxima First Strand cDNA Synthesis Kit (Fermentas), following the manufacturer’s recommendations. RT-qPCR was performed using SYBR Green PCR Master Mix (Fermentas) and specific primer sets for the gene of interest (Supplementary Table S1). The samples were run on a MX3000 quantitative PCR thermocycler (Stratagene) with an initial step at 95°C for 10 min, 40 cycles of 15 s at 95°C, 30 s at 60°C and 30 s at 72°C. Data was analyzed using the Relative Expression Software Tool – Multiple Condition Solver REST-MCS version 2 (Pfaffl, 2001; Pfaffl et al., 2002). The *tpi* and *rpoB* genes served as reference genes. The experiment was carried out in three biological replicates, each in technical duplicates. Statistical differences were analyzed with a randomization test provided in the REST software. Only differences with at least 95% confidence were reported as statistically significant.

### *In Silico* Predictions

The IntaRNA software (Busch et al., 2008; Wright et al., 2014; Mann et al., 2017) was used for predicting interactions between target mRNAs and sRNAs. Full length sequences of sRNAs and truncated versions of the targets were employed. Secondary structure predictions were obtained through Mfold (Zuker, 2003).

### Electrophoretic Mobility Shift Assays (EMSAs)

Templates for *in vitro* transcription carried a T7 RNA polymerase binding site at their 5′-end and were generated by PCR. Templates for *lmo0484* were obtained by PCR from chromosomal DNA and sRNA transcripts were made using overlapping primers (Supplementary Table S1). *In vitro* transcription, RNA purification, de-phosphorylation and labeling were performed as described previously (Sievers et al., 2014). EMSAs were performed as previously described (Nielsen et al., 2010). Briefly, 40 fmol of 5′-end labeled RNA was incubated with excess unlabeled RNA in a total volume of 10 μL in the presence of non-specific competitor (tRNA) for 1 h at 37°C followed by 10 min on ice. Samples were separated on a 5% non-denaturing gel at 4°C. RNA bands were visualized and analyzed as described for the northern blotting experiments.

### *In Vitro* Enzymatic Structure Probing

5′-end labeled transcripts were prepared as described for the EMSAs. The enzymatic probing was carried out as previously described (Sievers et al., 2014), with some deviations. Briefly, for the alkaline hydrolysis ladder, 0.2 pmol of labeled RNA was mixed with alkaline hydrolysis buffer (Ambion) and 10 μg of yeast tRNA (Ambion) in a total volume of 10 μL and incubated at 95°C for 5 min; for T1 control sample, 0.2 pmol of labeled RNA was denatured and incubated with 0.01 U of T1 RNase (Ambion) for 5 min. Structure probing RNA interactions were incubated at 37°C for 1 h before treating the samples with the indicated cleaving agent: 0.01 U T1 RNase for 5 min and 0.0015 U V1 RNase (Ambion) for 2 min. Control samples were prepared likewise (except for the cleaving agents) and incubated at 37°C for the duration of the experiment. Samples were placed on ice and mixed with 2× loading buffer type II (Ambion). Five μL of each sample was separated on an 8% denaturing polyacrylamide gel. RNA bands were visualized and analyzed as described for the northern blotting experiments.

## Results

### LhrC1–5 Are Highly Induced by Hemin in a LisR-Dependent Manner

**FIGURE 1:**
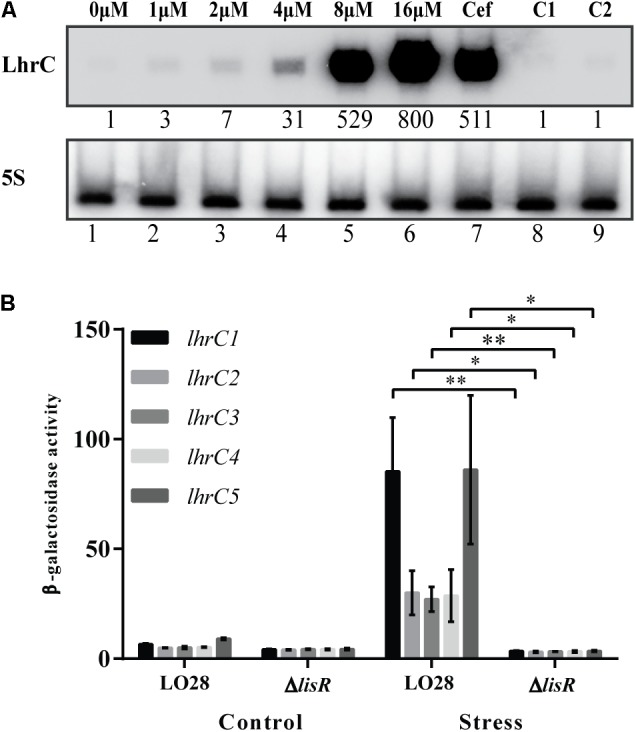
Induction of LhrC1–5 during hemin stress. **(A)** Northern blot analysis of LhrC1–5 expression. Samples were taken from *Listeria monocytogenes* LO28 wild-type cultures stressed with increasing concentrations of hemin (lanes 1–6), with a sub-inhibitory concentration of cefuroxime (9 μM) (lane 7) or with the hemin dissolvent NaOH (the same volume used to dissolve 8 and 16 μM hemin – lanes 8 and 9, respectively). Northern blot was probed for LhrC1–5 and 5S rRNA as a loading control. Relative levels of LhrC1–5 (normalized to 5S) are shown below each lane. **(B)** Transcriptional reporter gene fusions of *lhrC* promoters. Plasmids containing each of the five *lhrC* promoter regions fused to *lacZ* (Sievers et al., 2014) were transformed into LO28 wild-type and Δ*lisR*. The resulting strains were grown up to OD_600_ = 0.35 and stressed with hemin (8 μM), after control samples had been taken (Control). Further samples for a following β-galactosidase assay were withdrawn after 2 h (Stress). Results are the average of three biological replicates, each carried out in technical duplicates. After 2 h of stress, a significant difference between the Δ*lisR* mutant and wild-type cells was observed (^∗^*p* < 0.05, ^∗∗^*p* < 0.005).

To investigate if LhrC1–5 are induced in response to hemin exposure, the sRNA levels were determined via northern blot analysis on total RNA purified from *L. monocytogenes* LO28 wild-type cells subjected to various concentrations of hemin for 1 h. As a control, wild-type cells were exposed to a sub-inhibitory concentration of the cell wall-acting antibiotic cefuroxime (9 μM), which is already known as an inducer of LhrC1–5 (Sievers et al., 2014). As seen in **Figure 1A**, LhrC1–5 levels were strongly induced with increasing concentrations of hemin (0, 1, 2, 4, 8, and 16 μM) and this induction was not caused by the hemin dissolvent NaOH (C1 and C2). In addition, exposure to 8 μM hemin induced LhrC1–5 to the same extent as 9 μM cefuroxime (Cef). The novel members of the LhrC family, Rli22 and Rli33-1, were also investigated through the same means to assess their induction under hemin stress conditions (Supplementary Figure S1A). Rli22 appeared to be induced when *L. monocytogenes* was exposed to the highest concentrations of hemin (8 and 16 μM), whereas the expression of Rli33-1 remained constant under all conditions tested.

To further investigate the induction of LhrC1–5 by hemin, the promoter activity of the five *lhrC* copies was determined using transcriptional fusions of each promoter to the reporter gene *lacZ* in the vector pTCV-lac (Sievers et al., 2014). As the TCS LisRK has been shown to play a role in the activation of *lhrC1–5* under cell envelope stress conditions (Sievers et al., 2014), both the wild-type strain and a mutant strain lacking the response regulator LisR (Δ*lisR*) were transformed with the promoter-*lacZ* plasmids. The β-galactosidase activity was determined 2 h after subjecting the cultures to hemin stress (8 μM) and non-stressed cultures were included as controls (**Figure 1B**). In line with our previous observations, the promoter-*lacZ* constructs gave rise to β-galactosidase activity close to background levels under non-stress conditions (Sievers et al., 2014). However, after 2 h of hemin stress, a significant increase in the β-galactosidase activity was observed in the wild-type strain carrying the *lhrC-lacZ* fusion plasmids, while no increase in activity was detected in the Δ*lisR* cells. The activity of *rli22* and *rli33-1* promoters fused to *lacZ* was also determined in wild-type cells (Supplementary Figure S1B). Notably, the β-galactosidase activity in non-stressed and stressed cultures remained similar, showing that these promoters were not significantly activated by the addition of hemin. Overall, the results confirmed the induction of LhrC1–5 by hemin exposure and the requirement of LisR in the regulation of *lhrC1–5* when *L. monocytogenes* faces excess concentrations of hemin. In contrast, the effect of hemin on *rli22* and *rli33-1* appeared to be negligible, and for that reason the following experiments will only focus on LhrC1–5.

### LhrC1–5 and LisR Play a Role in the Adaptation to Hemin Toxicity

**FIGURE 2:**
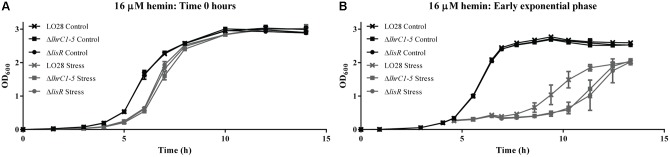
Stress tolerance assay. **(A)** LO28 wild-type, Δ*lhrC1–5* and Δ*lisR* strains were grown in BHI (Control) and BHI containing 16 μM hemin (Stress). **(B)** LO28 wild-type, Δ*lhrC1–5* and Δ*lisR* were grown in BHI to OD_600_ = 0.2. Then, the cultures were split in two; one half was stressed with 16 μM hemin (Stress) and the other half kept unstressed (Control). Bacterial growth was monitored until all cultures reached stationary phase. For each assay, the average of three biological replicates is shown.

To investigate whether LhrC1–5 and/or the response regulator LisR contribute to the adaptation of *L. monocytogenes* to hemin stress, growth of the wild-type strain and strains lacking LhrC1–5 or LisR was compared when these cultures were exposed to 16 μM hemin. Hemin was either added to the cultures at the beginning of the growth experiment (**Figure 2A**), or when the cells reached the early exponential phase (**Figure 2B**). No difference in growth was observed between the wild-type and the two mutant strains when hemin was added at time 0 h (**Figure 2A**). However, when hemin was added to exponentially growing cells, the strains clearly responded differently. As seen in **Figure 2B**, both mutant strains struggled to adapt to the stress condition compared to the wild-type, suggesting the involvement of LhrC1–5 and LisR in the adaptation of *L. monocytogenes* to excess hemin.

### LhrC1–5 Down-Regulate Their Known Target Genes Under Hemin Stress

**FIGURE 3:**
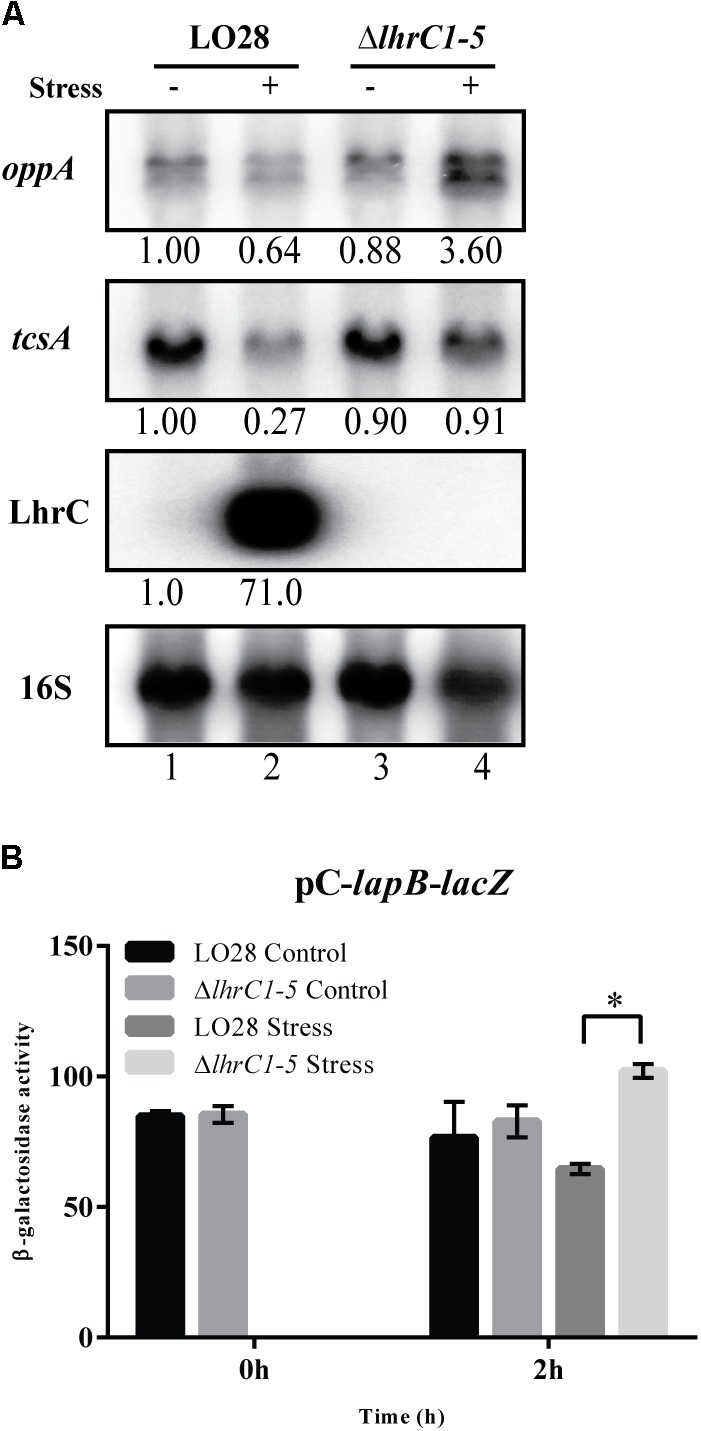
LhrC-mediated down-regulation of the known target genes *oppA*, *tcsA* and *lapB*. **(A)** Northern blot analysis of *oppA* mRNA, *tcsA* mRNA and LhrC1–5. Samples were taken from LO28 wild-type and Δ*lhrC1–5* cultures exposed to 8 μM hemin stress for 1 h (+) as well as from non-stressed cultures (–). Northern blots were probed for *oppA* mRNA, *tcsA* mRNA, LhrC1–5 and 16S rRNA (loading control). Relative levels of *oppA* mRNA, *tcsA* mRNA and LhrC1–5 (normalized to 16S) are shown below each lane. **(B)** β-galactosidase assay of LO28 wild-type and Δ*lhrC1–5* strains carrying a translational reporter gene fusion of *lapB* to *lacZ* in the vector pCK-lac (Sievers et al., 2014). β-galactosidase activities of wild-type and mutant cells were measured at the indicated time points under non-stress conditions (Control) and after exposure to 8 μM hemin (Stress). The results are the average of three biological replicates, each carried out in technical duplicates. After 2 h of stress, a significant difference (asterisk) between the mutant and wild-type cells was observed (*p* < 0.0001).

Three genes (*lapB*, *oppA,* and *tcsA*) were previously identified as direct targets of LhrC1–5 (Sievers et al., 2014, 2015). More specifically, LhrC1–5 down-regulate their expression at the post-transcriptional level in response to cefuroxime stress. This raises the question whether LhrC1–5 regulate the same set of targets, no matter the stress factor causing their induction, or if LhrC1–5 have different targets under different stress conditions. To investigate if LhrC1–5 control the expression of *oppA* and *tcsA* under hemin stress, we made use of northern blot analysis to assess the *oppA* and *tcsA* mRNA levels in wild-type and Δ*lhrC1–5* strains. The cultures were grown to early exponential phase and subjected to 8 μM hemin for 1 h; non-stressed cultures were included as controls (**Figure 3A**). In wild-type cells, hemin exposure resulted in a minor decrease in *oppA* mRNA (less than 1.5-fold), whereas for the *lhrC1–5* mutant strain, the *oppA* mRNA level was 4-fold higher, when comparing hemin stressed and non-stressed cells. For *tcsA*, a threefold decrease was seen when wild-type cells were subjected to hemin stress, whereas no major changes were observed when comparing the levels of *tcsA* mRNA in stressed and non-stressed cultures of the Δ*lhrC1–5* strain. Altogether, the northern blot analysis demonstrated that the induction of LhrC1–5 by hemin diminishes the expression of *oppA* and causes down-regulation of *tcsA* (**Figure 3A**). Strikingly, the regulatory effect observed for LhrC1–5 on *oppA* and *tcsA* is comparable to the one obtained when using cefuroxime as an inducer of LhrC1–5 (Sievers et al., 2015). Clearly, LhrC1–5 down-regulate the mRNA levels of *oppA* and *tcsA* to a similar extent in response to both cefuroxime and hemin stresses.

As *lapB* is part of a large operon, a reporter gene fusion strategy was used to assess the effect of hemin-induced LhrC sRNAs on this target gene (Sievers et al., 2014). In the pC-*lapB-lacZ* construct, a sequence including the 5′-untranslated region and the first codons of *lapB*’s coding region was fused downstream of a moderate promoter and inserted in-frame to *lacZ* in vector pCK-lac (Sievers et al., 2014). Notably, the moderate promoter was not affected by LhrC1–5 under hemin stress (Supplementary Figure S2A). The β-galactosidase activities were measured at time 0 and 2 h relative to the onset of hemin stress (8 μM), and non-stressed cultures were included as controls (**Figure 3B** and Supplementary Figure S2B). After hemin exposure, the Δ*lhrC1–5* cells containing pC-*lapB*-*lacZ* showed higher β-galactosidase activity relative to the wild-type cells, whereas no difference in activity was observed under non-stress (control) conditions. These results demonstrate that LhrC1–5 down-regulate this target gene at the post-transcriptional level in response to hemin stress.

### Proteins Related to Heme Uptake and Utilization Are Affected by LhrC1–5 Under Cefuroxime Stress

In a previous study, Sievers et al. (2015) performed transcriptomic and proteomic analyses of LO28 wild-type and Δ*lhrC1–5* cells to identify genes controlled by LhrC1–5. For the transcriptome study, the cultures were subjected to cefuroxime stress for 30 min to induce LhrC1–5 regulation, whereas 1 h of cefuroxime stress was chosen for the proteomic analysis. These studies generated two lists of genes that were significantly up- or down-regulated in the *lhrC1–5* mutant strain relative to the wild-type at the RNA and protein levels, respectively. By then, tight parameters were employed to select the genes to be further characterized as potential targets of LhrC1–5. Thus, only genes that were regulated at least 1.5-fold by LhrC1–5 at the RNA level and 2.0-fold at the protein level in all three biological replicates were chosen for further investigation (Sievers et al., 2015). Only three genes passed these strict criteria, including *oppA* and *tcsA*. Finding that LhrC1–5 also act as regulatory sRNAs in response to hemin stress prompted us to search these lists for potential targets related to heme uptake and utilization. Indeed, when searching through the data obtained from the proteomic analysis, we found that Lmo0484 was more than threefold up-regulated in Δ*lhrC1–5* relative to the wild-type strain in all three biological replicates (Sievers et al., 2015). Lmo0484 is a homolog of the IsdG heme oxygenase from *S. aureus*, which degrades exogenous heme in the cytoplasm, leading to the release of free iron to be used as a nutrient source (Wu et al., 2005). In addition, we found that in two of the three biological replicates, the proteins Lmo2186 and Lmo2185 were more than three and twofold up-regulated, respectively, in the Δ*lhrC1–5* strain relative to wild-type (Sievers et al., 2015). Lmo2186 and Lmo2185 are homologs of IsdC from *S. aureus* (Newton et al., 2005) and were previously characterized as heme-binding proteins 1 and 2 (Hbp1 and Hbp2), respectively (Xiao et al., 2011). Even though none of these genes were significantly affected by LhrC1–5 at the RNA level following 30 min of cefuroxime stress (Sievers et al., 2015), the results obtained from the proteomic analysis made it relevant to further investigate the regulatory effect of LhrC1–5 on *lmo0484*, *lmo2186,* and *lmo2185*.

**FIGURE 4:**
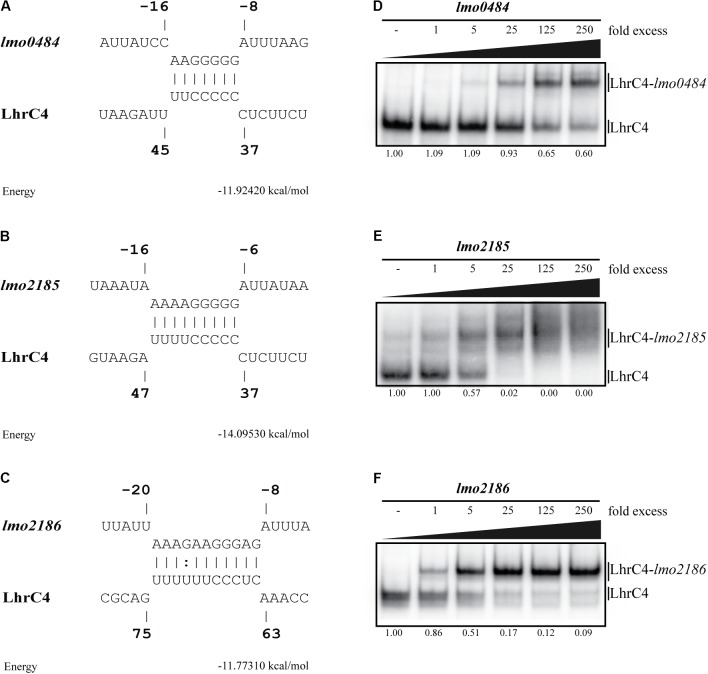
Analyzing the sRNA-mRNA interaction between LhrC4 and *lmo0484*, *lmo2185,* or *lmo2186*. **(A–C)** *In silico* prediction of sRNA-mRNA interactions. According to the IntaRNA Software (Busch et al., 2008; Wright et al., 2014; Mann et al., 2017), the loop A of LhrC4 binds to **(A)** *lmo0484* and **(B)** *lmo2185* mRNAs, and the single-stranded stretch binds to **(C)** *lmo2186* mRNA. All the interactions are predicted to block the SD sequence of the mRNAs. LhrC4 is shown as a representative of the five LhrC copies. The nucleotides of *lmo0484*, *lmo2185,* and *lmo2186* are numbered relative to the translation start site, and the nucleotides of LhrC4 are numbered relative to the 5′-end of the sRNA. **(D–F)** Testing the formation of sRNA-mRNA complexes by EMSAs. Labeled LhrC4 was shifted with increasing concentrations of unlabeled **(D)** *lmo0484* RNA, **(E)** *lmo2185* RNA or **(F)** *lmo2186* RNA. Fold excess refers to the amount of unlabeled mRNA added to each sample, relative to the amount of labeled LhrC4. The fraction of unbound LhrC4 is shown below each lane.

In regard to the known mode of action of LhrC1–5, we hypothesized that the sRNAs might act on *lmo0484*, *lmo2186,* and *lmo2185* via direct binding to the mRNAs, leading to inhibition of translation initiation. To investigate this assumption, we first performed *in silico* analyses of the potential basepairing between the sRNAs and mRNAs. Using IntaRNA (Busch et al., 2008; Wright et al., 2014; Mann et al., 2017), we predicted that LhrC1–5 could bind to the SD region of *lmo0484*, *lmo2185,* and *lmo2186* mRNAs (**Figures 4A–C** and Supplementary Figure S3). To verify experimentally the binding of the LhrCs to these mRNAs, EMSAs were performed, where LhrC4 was used as a representative of the LhrC family (**Figures 4D–F**). 5′-end labeled LhrC4 was visibly able to bind all three RNAs, where a single shifted band appeared with increasing concentrations of unlabeled *lmo0484*, *lmo2185,* or *lmo2186* RNA. These results clearly demonstrate that LhrC4 interacts with these mRNAs (**Figure 4**).

### LhrC4 Loop A Binds to the SD Region of *lmo0484* mRNA

**FIGURE 5:**
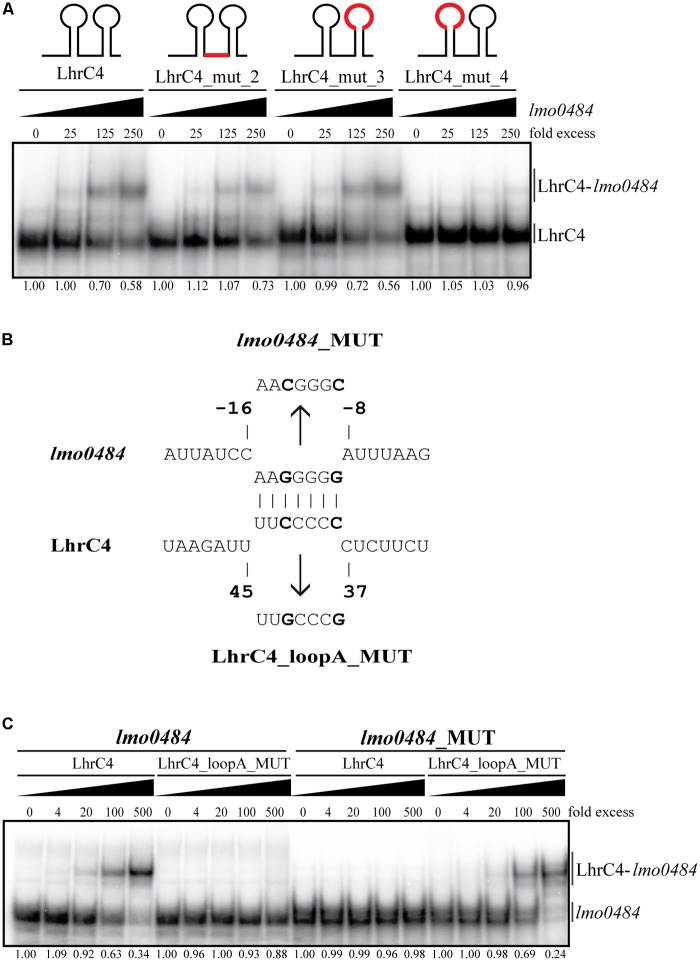
Electrophoretic mobility shift assays (EMSAs) of the interaction between LhrC4 and *lmo0484* mRNA. **(A)** LhrC4 mutant screening of loop A, single-stranded stretch, and the terminator loop. Labeled LhrC4 and the mutant derivatives were tested for their ability to interact with unlabeled *lmo0484* RNA. The mutated regions are shown in red in the sRNA sketches. LhrC4: wild-type LhrC4; LhrC4_mut_2: mutation in single-stranded stretch; LhrC4_mut_3: mutation in terminator loop; LhrC4_mut_4: mutation in loop A. The fraction of unbound LhrC4 is shown below each lane. **(B)** Predicted basepairing between the SD region of *lmo0484* mRNA and loop A of LhrC4. The mutated nucleotides are shown in bold and the sequences of the minimal mutant variants *lmo0484*_MUT and LhrC4_loopA_MUT are indicated. **(C)** Labeled *lmo0484* RNA and *lmo0484*_MUT were each incubated with increasing concentrations of unlabeled LhrC4 or the mutant variant LhrC4_loopA_MUT. The fraction of unbound *lmo0484* RNA is shown below each lane.

Based on the results obtained from the proteomic analysis and the *in vitro* binding studies, we decided to further analyze the basepairing between the sRNA LhrC4 and *lmo0484* mRNA. All five LhrCs hold three single-stranded CU-rich regions known to interact with their target mRNAs (Sievers et al., 2014): loop A, a single-stranded stretch and the terminator loop. To investigate the importance of the CU-rich regions for the interaction with *lmo0484* mRNA, mutant versions of LhrC4, where the entire CU-rich region is mutated, were tested for their ability to bind wild-type *lmo0484* in an EMSA [for details about the mutations, see Sievers et al. (2014)]. The LhrC4 mutant derivatives were labeled and mixed with increasing concentrations of *lmo0484* RNA (**Figure 5A**). The results revealed that mutations in the single-stranded stretch (LhrC4_mut_2) and in the terminator loop (LhrC4_mut_3) did not reduce the interaction between the two RNAs, whereas the loop A mutation (LhrC4_mut_4) abolished the basepairing, suggesting that this region is crucial for the binding of LhrC4 to *lmo0484* (**Figure 5A**). To investigate if the SD region of *lmo0484* is responsible for basepairing to LhrC4 loop A, *lmo0484* was mutated in two of the seven nucleotides in the predicted LhrC4 binding region (**Figure 5B**). Interestingly, the mutated version of *lmo0484* (*lmo0484*_MUT) was not able to bind the wild-type version of LhrC4 (**Figure 5C**). Finally, we created a mutant version of LhrC4 containing the complementary nucleotide mutations of *lmo0484*_MUT in loop A (LhrC4_loopA_MUT) (**Figure 5B**). As expected, the LhrC4_loopA_MUT variant was unable to bind the wild-type version of *lmo0484*, but when testing the binding of LhrC4_loopA_MUT to *lmo0484*_MUT, the interaction was restored (**Figure 5C**), confirming the importance of loop A for basepairing to the SD region of *lmo0484* mRNA.

**FIGURE 6:**
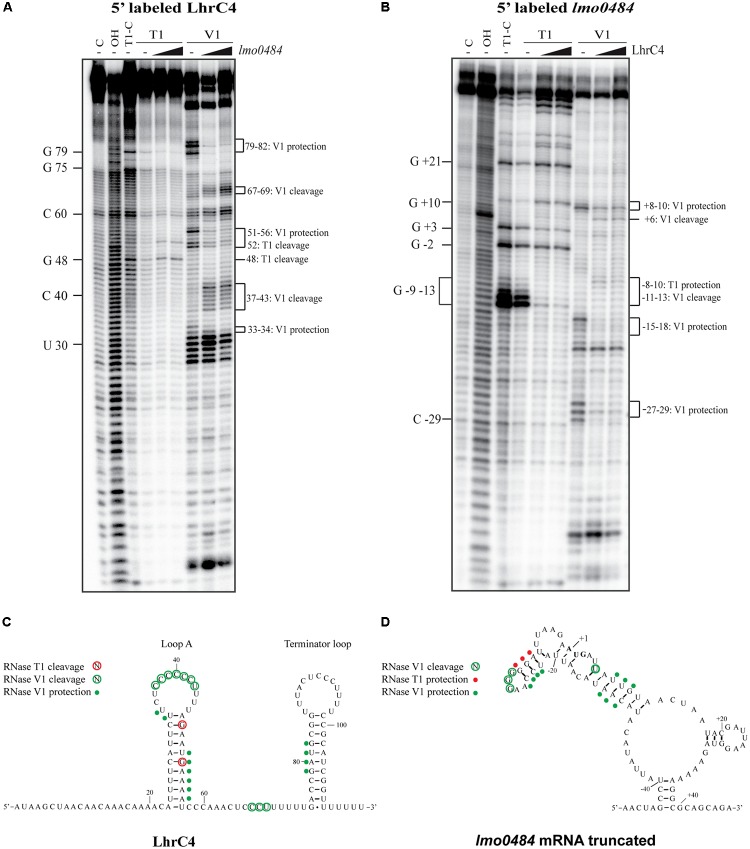
Structure probing of LhrC4 and *lmo0484* mRNA interaction. **(A)** 5′-end labeled LhrC4 was treated with RNase T1 or RNase V1, either in the absence (–) or in the presence of 25- or 250-fold excess of unlabeled *lmo0484* RNA. As a control, untreated LhrC4 was separated in the first lane (C), an alkaline ladder (OH) is shown in the second lane and an RNase T1 ladder (T1-C) was separated in the third lane. For an overview, selected nucleotides are labeled on the left side. Nucleotides showing structural changes upon *lmo0484* binding are marked on the right side of the gel. **(B)** Partial digestion of 5′-end labeled *lmo0484* RNA with RNases T1 and V1. Untreated *lmo0484* RNA (C), an alkaline ladder (OH) and an RNase T1 ladder (T1-C) are shown in the first three lanes. Some of the cleaved G residues are labeled along the left side of the gel. The *lmo0484* nucleotides, that were protected or cleaved in the presence of LhrC4, are indicated on the right side of the gel. **(C)** Secondary structure of LhrC4 illustrating the cleavage pattern upon binding to *lmo0484* mRNA. Residues cleaved by RNase T1 (red) or RNase V1 (green) are encircled. Residues of LhrC4, that appeared to be protected by *lmo0484* RNA, are indicated by green dots. The nucleotides of LhrC4 are numbered relative to the 5′-end of the sRNA. **(D)** Secondary structure of the truncated version of *lmo0484* mRNA showing an altered cleavage pattern upon the addition of LhrC4. Residues cleaved by RNase V1 (green) are encircled. Residues of *lmo0484* RNA that were protected in the presence of LhrC4 are indicated by red dots (T1 protection) or green dots (V1 protection). The start codon is indicated in bold. The nucleotides of *lmo0484* are numbered relative to the translation start site (+1).

To study the structural implications of the interaction between the two RNAs, structural probing was employed. First, 5′-end labeled LhrC4 was subjected to RNase hydrolysis in the absence and presence of unlabeled *lmo0484* RNA (**Figure 6A**). The enzymes RNase T1 and RNase V1 were used to cleave the RNAs, where the former is specific for unpaired guanine and the latter for double-stranded regions. The region from nucleotide 37 to 43, which resides in loop A, presented an increased V1 cleavage when LhrC4 interacts with *lmo0484* (**Figures 6A,C**). Thus, the nucleotides residing in loop A went from single-stranded to double-stranded upon binding to *lmo0484*, confirming the importance of loop A for the sRNA-mRNA interaction. The V1 protection of nucleotides 51–56 and the increased T1 cleavage of G48 and G52 suggest that binding of loop A to *lmo0484* disrupts the double-stranded structure of stem A. An increased V1 cleavage was also seen at nucleotides 67–69, corresponding to the single-stranded stretch in LhrC4, indicating that this CU-rich region may play a role in the basepairing of LhrC4 to *lmo0484* as well, but to a lesser extent than loop A. Similarly, the region from nucleotides 79–82, residing in the double-stranded region of the terminator loop, obtained a more single-stranded conformation (V1 protection) in the presence of *lmo0484*, suggesting an opening of the terminator stem upon basepairing between the target mRNA and the single-stranded stretch (**Figures 6A,C**). To corroborate these results, 5′-end labeled *lmo0484* RNA was subjected to the action of the same RNases in the absence and presence of unlabeled LhrC4 (**Figure 6B**). The nucleotides from -8 to -13, corresponding to the putative SD region of *lmo0484*, changed from single-stranded to double-stranded conformation (from -8 to -10: increased cleaving by V1; from -11 to -13: protection from T1) (**Figures 6B,D**). This result clearly supports that the SD region is bound by LhrC4. Additionally, some nucleotides shifted from double-stranded to single-stranded conformations (-27 to -29, -15 to -18 and +8 to +10), most likely reflecting changes in the secondary structure of *lmo0484* occurring as a consequence of LhrC4 basepairing to the SD region (**Figures 6B,D**). Collectively, the results of the EMSAs and structure binding experiments support that the CU-rich region of loop A, and to a lesser extent the single-stranded stretch, binds to the SD region of *lmo0484* mRNA.

### LhrC1–5 Control the Expression of *lmo0484* at the Post-transcriptional Level in Response to Cefuroxime Stress

**FIGURE 7:**
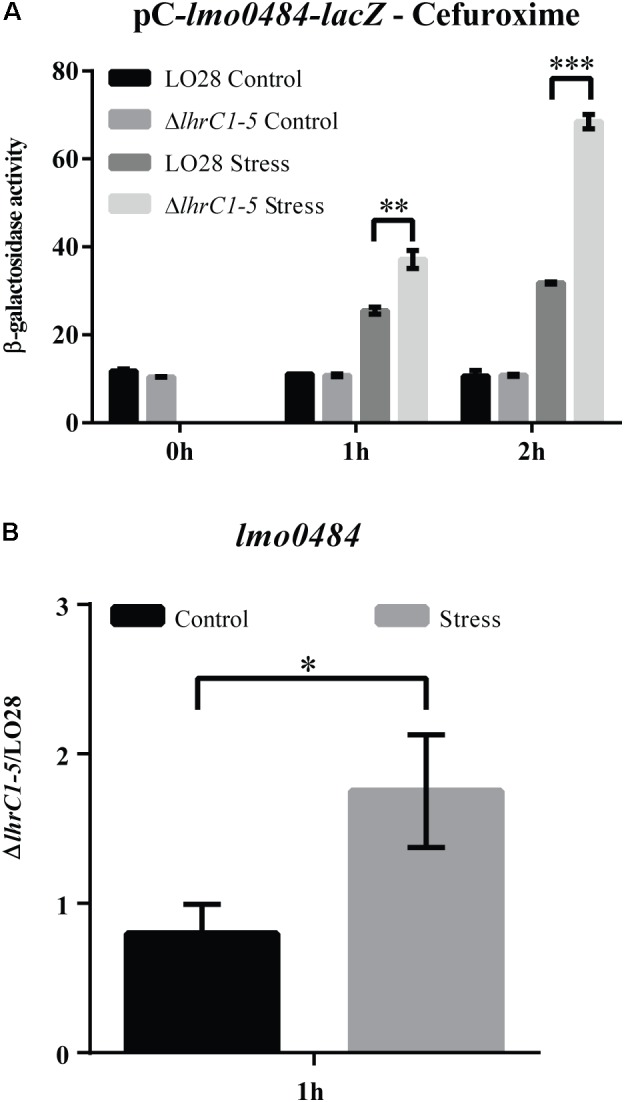
Assessing the effect of LhrC1–5 on the novel target gene *lmo0484* upon cefuroxime exposure. **(A)** β-galactosidase assay of LO28 wild-type and Δ*lhrC1–5* strains carrying a translational reporter gene fusion of *lmo0484* to *lacZ* in the vector pCK-lac. β-galactosidase activities of LO28 wild-type and mutant cells were measured at the indicated time points under non-stress conditions (Control) and after exposure to 9 μM cefuroxime (Stress). The results are the average of three biological replicates, each carried out in technical duplicates. After 1 and 2 h of stress, a significant difference between the mutant and wild-type cells was observed (^∗∗^*p* < 0.001; ^∗∗∗^*p* < 0.0001). **(B)** Quantification of *lmo0484* mRNA in Δ*lhrC1–5* relative to LO28 wild-type by RT-qPCR. The ratio of Δ*lhrC1–5*/LO28 was determined at the 1 h time point for both non-stressed (Control) and cefuroxime-exposed samples (Stress). The result shown is the average of three biological replicates. The asterisk indicates a significant increase of the ratio under stress conditions compared to the control with *p* < 0.05.

After showing that LhrC4 basepairs to the SD region of *lmo0484* mRNA *in vitro*, we aimed to test the mechanism by which LhrC1–5 regulate this target *in vivo*. To this end, we analyzed the regulatory effect of LhrC1–5 at the post-transcriptional level on *lmo0484* by making use once again of the *lacZ* reporter strategy. First, the 5′-end of the *lmo0484* transcript was mapped by primer extension analysis to position -48 relative to the translation start site (Supplementary Figure S4). Then, a sequence encoding the 5′-end of the *lmo0484* transcript and additional 47 bp of *lmo0484* coding region was fused downstream of a moderate promoter and fused in-frame to *lacZ* in the vector pCK-lac (Nielsen et al., 2010). The generated pC-*lmo0484*-*lacZ* construct was transformed into both wild-type and Δ*lhrC1–5* cells. The resulting strains were then subjected to cefuroxime stress for 1 or 2 h, and the β-galactosidase activity was measured. During cefuroxime stress, higher activity levels were obtained in Δ*lhrC1–5* cells compared to wild-type cells, confirming that LhrC1–5 down-regulate the expression of *lmo0484* at the post-transcriptional level in response to cefuroxime exposure (**Figure 7A** and Supplementary Figure S2C).

As *lmo0484* was not significantly affected by LhrC1–5 at the RNA level after 30 min of cefuroxime stress (Sievers et al., 2015), we performed RT-qPCR on total RNA purified from cells exposed to cefuroxime for 1 h, where the level of LhrC1–5 induction is known to be at its highest (Sievers et al., 2014); non-stressed samples were included as controls. The *lmo0484* mRNA level was quantified in Δ*lhrC1–5* cells relative to wild-type cells under non-stress (control) and stress conditions (**Figure 7B**). In the control samples, the mRNA ratio was approximately 1, showing that there was no difference in *lmo0484* expression in the wild-type and mutant strains grown under non-stress conditions. In contrast, *lmo0484* mRNA expression was 1.75-fold higher in Δ*lhrC1–5* compared to wild-type after 1 h of cefuroxime stress. Collectively, the results of the β-galactosidase assay and the RT-qPCR experiment demonstrate that LhrC1–5 down-regulate *lmo0484* expression at the post-transcriptional level upon subjecting *L. monocytogenes* to cefuroxime stress. Thus, *lmo0484* is clearly a target for LhrC1–5-mediated control when *L. monocytogenes* is exposed to cell envelope stress conditions.

### *lmo0484* Is Strongly Repressed in Response to Hemin Exposure and Is Dispensable for Growth Under Hemin Stress Conditions

**FIGURE 8:**
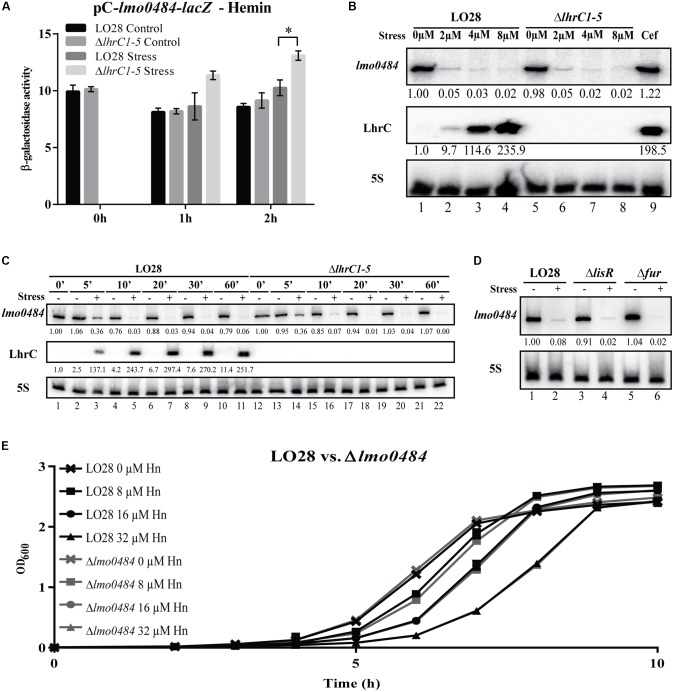
Assessing the effect of LhrC1–5 on the novel target gene *lmo0484* under hemin stress. **(A)** β-galactosidase assay of LO28 wild-type and Δ*lhrC1–5* strains carrying a translational reporter gene fusion of *lmo0484* to *lacZ* in the vector pCK-lac. β-galactosidase activities of wild-type and mutant cells were measured at the indicated time points under non-stress conditions (Control) and after exposure to 8 μM hemin (Stress). The results are the average of three biological replicates, each carried out in technical duplicates. After 2 h of stress, a significant difference between the mutant and wild-type cells was observed (*p* < 0.005). **(B)** Northern blot analysis of *lmo0484* mRNA after exposure to various concentrations of hemin. Samples were taken from LO28 wild-type and Δ*lhrC1–5* cultures exposed to 1 h of 0, 2, 4, or 8 μM hemin stress. A sample from LO28 wild-type culture exposed to 9 μM cefuroxime was used as a control. Northern blots were probed for *lmo0484* mRNA, LhrC1–5 and 5S rRNA (loading control). Relative levels of *lmo0484* mRNA and LhrC1–5 (normalized to 5S) are shown below each lane. **(C)** LhrC induction profile and time-dependent regulation of *lmo0484* mRNA in response to hemin stress. At time = 0, corresponding to OD_600_ = 0.35, LO28 wild-type and Δ*lhrC1–5* cells were split in two, and one half of the culture was treated with 8 μM hemin (+), whereas the other half was left untreated (–). Samples were harvested at several time points (0, 5, 10, 20, 30, and 60 min relative to the addition of hemin) and total RNA was prepared for northern blot analysis. The blot was probed for *lmo0484* mRNA, LhrC1–5 and 5S rRNA (loading control). Relative levels of *lmo0484* mRNA and LhrC1–5 were normalized to 5S and are shown below each lane. **(D)** Testing the role of LisR and Fur in the heme-dependent repression of *lmo0484*. Samples were taken from LO28 wild-type, Δ*lisR* and Δ*fur* cultures exposed for 1 h to 8 μM hemin stress (+) as well as from non-stressed cultures (–). Northern blots were probed for *lmo0484* mRNA and 5S rRNA (loading control). Relative levels of *lmo0484* mRNA normalized to 5S are shown below each lane. **(E)** Stress tolerance assay of the Δ*lmo0484* strain compared to LO28 wild-type. Wild-type and Δ*lmo0484* strains were grown in BHI containing 0, 8, 16, or 32 μM hemin. Bacterial growth was monitored until all cultures reached stationary phase. The average of three biological replicates is shown.

Based on the results obtained so far, we reasoned that the LhrCs could down-regulate the expression of *lmo0484* in response to hemin stress. To test this hypothesis, wild-type and Δ*lhrC1–5* cells containing the pC-*lmo0484*-*lacZ* plasmid were exposed to hemin stress for 1 and 2 h, and non-stressed cultures were used as controls. Upon the addition of hemin, the β-galactosidase activity in the wild-type and Δ*lhrC1–5* cells remained at relatively low levels (**Figure 8A** and Supplementary Figure S2D), which is in stark contrast to the increase in *lmo0484-lacZ* expression observed upon cefuroxime exposure (**Figure 7A**). After 2 h of hemin stress, the *lmo0484-lacZ* expression was only slightly, but significantly higher in the Δ*lhrC1–5* cells relative to the wild-type cells (**Figure 8A**). These results prompted us to investigate the effect of hemin stress on *lmo0484* at the RNA level. First, the expression of *lmo0484* was investigated in wild-type and Δ*lhrC1–5* cells subjected to increasing concentrations of hemin for 1 h (**Figure 8B**). The results of the northern blot experiment clearly demonstrated a strong repression of *lmo0484* transcript levels in the wild-type strain already at the lowest concentration of hemin tested. For comparison, the *lmo0484* mRNA level in the wild-type strain remained unaffected by 9 μM cefuroxime (Cef). When comparing the wild-type and Δ*lhrC1–5* cells after 1 h of hemin exposure, a regulatory effect of LhrC1–5 on *lmo0484* was not evident, although LhrC1–5 was clearly expressed (**Figure 8B**).

To further explore the response of *L. monocytogenes* to excess hemin, a time course experiment was performed. Using northern blot analysis, the levels of LhrC1–5 and *lmo0484* mRNA were determined in wild-type and Δ*lhrC1–5* cells at various time points after hemin exposure (**Figure 8C**). In the wild-type background, LhrC1–5 were clearly detected just 5 min after the addition of hemin, reaching a peak after 20 min of stress. A strong decrease in the transcript level of *lmo0484* was observed after 5 min of stress, demonstrating that *L. monocytogenes* responds very quickly to excess hemin by shutting down the expression of *lmo0484* mRNA. Once again, no major differences were observed when comparing the *lmo0484* mRNA levels in the wild-type and Δ*lhrC1–5* strains, suggesting that additional regulatory factors are involved in mediating the repression of *lmo0484* (**Figure 8C**). Indeed, a Fur box is present upstream from *lmo0484* (Supplementary Figure S4), suggesting that the iron-responsive regulator Fur could be a negative regulator of *lmo0484* expression. To investigate this, the *lmo0484* levels were tested in a mutant strain lacking the Fur regulator (**Figure 8D**). The results clearly demonstrate that Fur is not responsible for down-regulating the expression of *lmo0484* in response to hemin stress, and LisR seems not to be required either. Curiously, *lmo2185* and *lmo2186* transcript levels in the wild-type strain were also strongly repressed by the lowest concentration of hemin tested, and similarly, Fur was not responsible for the down-regulation observed (Supplementary Figures S4, S5).

In Gram-positive bacteria, members of the IsdG family are known to be required for the utilization of heme as an iron source (Reniere et al., 2010; Sheldon and Heinrichs, 2015). In addition, IsdG in *Bacillus anthracis* also acts to protect the bacterium against heme-mediated toxicity (Skaar et al., 2006). To assess the role of *lmo0484* during growth of *L. monocytogenes* under hemin stress conditions, a mutant strain lacking *lmo0484* was constructed. No significant difference was observed when comparing the growth of the Δ*lmo0484* and wild-type strains under hemin stress (**Figure 8E**).

To summarize, the mRNA level of *lmo0484* is quickly diminished upon hemin exposure by an unknown mechanism. Likewise, the level of *lmo2186-2185* mRNA is strongly reduced under hemin-rich conditions. It is possible that LhrC1–5 act to ensure an efficient shut-down of the translation of the few *lmo0484* and *lmo2186-2185* mRNAs remaining after the onset of hemin exposure. In accordance with the expression profile of *lmo0484* upon hemin exposure, this IsdG-encoding gene does not contribute to growth under hemin stress conditions.

## Discussion

Bacteria are known to utilize heme as an iron source during infection, however, in heme-rich environments, such as in the bloodstream and blood-rich organs, successful pathogens must defend themselves against the harmful effects of heme toxicity (Choby and Skaar, 2016; Joubert et al., 2017). Indeed, during severe hemolysis, free heme may reach concentrations up to 20 μM (Arruda et al., 2004). Information on how *L. monocytogenes* maintains heme homeostasis is relatively limited and biased toward studies on the acquisition of hemoglobin and heme under iron-limited conditions (Klebba et al., 2012; Lechowicz and Krawczyk-Balska, 2015). In this study, we focused on how *L. monocytogenes* senses and responds to excess heme. We report that heme generates a signal that stimulates LisR-mediated activation of members of the LhrC family of sRNAs. Furthermore, we show that LisR and LhrC1–5 contribute to the adaptation of *L. monocytogenes* to growth under conditions of excess heme. LisRK has previously been found to be important for infection in mice (Cotter et al., 1999; Kallipolitis and Ingmer, 2001), and, together with LhrC1–5, LisR contributes to the intracellular replication of *L. monocytogenes* in macrophage-like cells (Sievers et al., 2015). The expression of LhrC1–5 is known to be highly induced when *L. monocytogenes* is exposed to whole human blood (Toledo-Arana et al., 2009), which corresponds well with a role for the LisR-regulated sRNAs in the adaptation to heme-rich environments.

We have previously shown that LhrC1–5 are induced in a LisR-dependent manner under cell envelope stress conditions, such as exposure to the cell wall-acting antibiotic cefuroxime, and that LhrC1–5 down-regulate the expression of cell envelope-associated proteins with virulence functions (Sievers et al., 2014, 2015). In the present study, we show that LhrC1–5 also repress the expression of the three known target genes in response to heme toxicity, suggesting that a fine-tuning of the levels of the cell envelope-associated proteins TcsA, OppA, and LapB is beneficial for *L. monocytogenes* under heme-rich conditions. Notably, whole human blood contains multiple components participating in host immunity, and surface exposed proteins are readily recognized by the immune system (Sievers et al., 2015). Thus, in heme-rich conditions, such as the human blood, LhrC-mediated down-regulation of surface exposed proteins may be viewed as an attempt by *L. monocytogenes* to evade detection by the immune system. Interestingly, the present study suggests that genes encoding cell envelope-associated proteins with known functions in heme acquisition belong to the LhrC regulon as well. The LhrC4 sRNA bound readily to the SD regions of *lmo2186*-*lmo2185* mRNA, encoding the heme uptake proteins Hbp1 and Hbp2, respectively. Additionally, the *lmo0484* gene, encoding a heme oxygenase, was found to be a target for LhrC regulation. The LhrC sRNAs were shown to down-regulate the expression of *lmo0484* in response to cell envelope stress and LhrC4 was found to bind specifically to the SD region of *lmo0484* mRNA using one of its three CU-rich regions. These observations suggest that the sRNAs act to inhibit translation initiation of *lmo0484* under LhrC-inducing conditions, such as upon exposure to cefuroxime or heme. Whereas LhrC-mediated down-regulation of *lmo0484* expression was clearly detected in response to cefuroxime stress, the regulatory effect of LhrC1–5 on *lmo0484* was less pronounced under heme stress due to a rapid decrease in *lmo0484* mRNA levels following the addition of excess heme to the growth medium. Interestingly, a similar decrease was observed when testing the level of *lmo2186*-*lmo2185* mRNA upon heme exposure, suggesting a common mechanism for down-regulation of genes involved in heme uptake and utilization in response to heme stress. Although potential Fur boxes were found in the promoter regions upstream from *lmo2186-lmo2185* and *lmo0484*, the Fur regulator was not required for the heme-mediated repression. Thus, the mechanism underlying the repressive effect of excess heme on the heme uptake and utilization genes in *L. monocytogenes* remains elusive. Considering the potential damaging effects of heme, an instant shut-down of the heme uptake genes *lmo2186* and *lmo2185* in response to excess heme seems logical, whereas the repression of the heme oxygenase-encoding gene *lmo0484* is more ambiguous. In other organisms, including the bacterial pathogen *B. anthracis*, heme oxygenases have been shown to contribute to the heme detoxification process (Skaar et al., 2006), but in *L. monocytogenes*, the heme oxygenase encoded by *lmo0484* appears to be dispensable in heme-rich environments. Indeed, *L. monocytogenes* wild-type and Δ*lmo0484* strains were found to grow equally well under heme stress conditions (**Figure 8E**). Whether the second heme oxygenase encoded by *L. monocytogenes* (Isd-LmHde) contributes to the heme detoxification process remains to be investigated.

**FIGURE 9:**
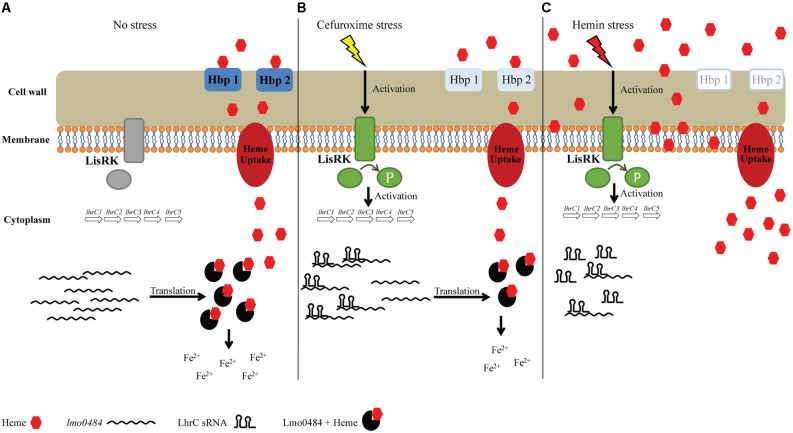
Proposed model of the regulatory effect of LhrC1–5 on heme uptake and utilization genes. **(A)** During growth in BHI medium, there is no activation of the TCS LisRK and subsequently LhrC1–5 are not induced. Under this condition, *lmo0484*, *lmo2186,* and *lmo2185* are expressed and heme-uptake from the BHI medium is taking place according to the cells’ needs. The *lmo0484* mRNA is translated into a IsdG-like heme oxygenase, which will catalyze the degradation of heme to obtain free iron. **(B)** When the cells are subjected to cefuroxime stress, LisRK will be activated. LhrC1–5 are then induced and can bind *lmo0484* mRNAs. Binding of LhrC sRNA to the SD region of *lmo0484* mRNA leads to inhibition of translation. Under cefuroxime stress, LhrC1–5 most likely act to control the expression of the heme uptake genes *lmo2186* and *lmo2185* in a similar fashion (not shown). Thus, when exposed to cefuroxime, LhrC1–5 act to fine-tune the expression of heme uptake and utilization genes, suggesting a need for *L. monocytogenes* to slightly reduce the uptake of heme when the cell envelope is being stressed. **(C)** Under hemin stress, LhrC1–5 are strongly induced as well in a LisR-dependent manner. Furthermore, when the cell faces high concentrations of heme, the mRNA levels of *lmo0484* are almost immediately diminished by an unknown mechanism. In this situation, LhrC1–5 will bind the remaining *lmo0484* mRNAs in the cell and act as ‘vacuum cleaners’ to avoid translation of the residual *lmo0484* mRNA. The heme uptake genes *lmo2185* and *lmo2186* are most likely controlled by heme and LhrC1–5 in a similar fashion (not shown). Consequently, the expression of heme acquisition genes is quickly reduced in response to heme stress to avoid further uptake of heme and liberation of iron.

Based on our findings, we propose the following model for LhrC-mediated control of the heme uptake and utilization genes *lmo0484*, *lmo2186,* and *lmo2185* under cefuroxime stress and heme stress (see **Figure 9**): under non-stress conditions, these genes are clearly expressed (**Figures 8B–D** and Supplementary Figure S5), indicating that *L. monocytogenes* is using the heme already present in the BHI medium as a source of iron (**Figure 9A**); in response to cefuroxime stress, LhrC1–5 fine-tune the expression of *lmo0484*, and most likely also *lmo2186* and *lmo2185*, suggesting that *L. monocytogenes* is employing sRNA-mediated control to maintain heme homeostasis under cell envelope stress conditions (**Figure 9B**); upon exposure to excess heme, the levels of *lmo0484* and *lmo2186-lmo2185* mRNAs are strongly reduced by an unknown mechanism (e.g., heme-induced transcriptional repression and/or mRNA degradation) (**Figure 9C**). In this case, we speculate that the role of LhrC1–5 is to prevent translation of the remaining *lmo0484* mRNA, and possibly also *lmo2186-lmo2185* mRNA, following heme exposure. Notably, LhrC1–5 were induced more than 50-fold after just 5 min of heme stress, showing that the LhrC sRNAs are available for basepairing to target mRNAs within a few minutes (**Figure 8C**). Together, these regulatory mechanisms act to repress the expression of genes involved in heme uptake and utilization, which allows *L. monocytogenes* to adapt very quickly to heme-rich conditions. Collectively, this study shows that the detected outcome of LhrC-mediated control relies not only on the presence of the sRNAs, but also on the availability of the partner mRNAs, which may be subject to control by other regulatory mechanisms under specific stress conditions (**Figure 9C**).

The results obtained in this study clearly demonstrate a role for LisRK in the response to excess heme. In addition to LisRK, at least one more TCS is expected to contribute to the adaptation of *L. monocytogenes* to heme-rich conditions. In Gram-positive pathogens, the TCS HssRS has been shown to play a major role in the response to heme stress (Choby and Skaar, 2016). In *S. aureus* and *B. anthracis*, HssRS controls the expression of genes encoding a heme-regulated transporter, HrtAB, which protects the bacteria from heme toxicity by exporting heme (Torres et al., 2007; Stauff and Skaar, 2009). The HssRS system and HrtAB exporter are conserved in *L. monocytogenes* as well (Torres et al., 2007), however, their roles in dealing with heme toxicity remains to be clarified. Curiously, the HssRS system of *B. anthracis* was recently found to interact with another TCS, HitRS, which responds to compounds that affect the cell envelope integrity (Mike et al., 2014). HssRS and HitRS both act to stimulate *hrtAB* expression in response to heme and cell envelope stress, respectively. Furthermore, the histidine kinase HssS was shown to cross-phosphorylate the response regulator HitR to activate the expression of the *hitPQRS* operon upon heme exposure (Mike et al., 2014). This operon encodes components of an unstudied ABC transporter and the HitRS system. The cross-regulation between HssRS and HitRS is thought to ensure a coordinated response to heme and cell envelope stress in *B. anthracis*, which may enable this pathogen to better adapt and survive during infection (Mike et al., 2014). We found that heme stimulates LisR-dependent activation of LhrC1–5 as efficiently as the cell-wall acting antibiotic cefuroxime, revealing a link between the response to heme toxicity and cell envelope stress in *L. monocytogenes* as well. Future studies should focus on clarifying the interconnections (if any) between LisRK and the putative heme-responsive HssRS system in *L. monocytogenes.* Most importantly, it should be investigated if LisRK cross-phosphorylates with HssRS, as shown for HitRS and HssRS in *B. anthracis* (Mike et al., 2014).

In addition to the LhrCs in *L. monocytogenes*, a heme-responsive sRNA has also been described in *Pseudomonas aeruginosa*. The sRNA PrrH is encoded from the *prrF* locus in *P. aeruginosa* and overlaps with two iron-regulated sRNAs, PrrF1, and PrrF2 (Oglesby-Sherrouse and Vasil, 2010). In contrast to LhrC1–5, which are highly induced in response to excess heme, the PrrH sRNA is repressed by heme via an unknown mechanism. Furthermore, PrrH is repressed by iron, most likely via the Fur protein in *P. aeruginosa*. Although PrrH has been predicted to regulate genes involved in heme homeostasis and virulence through the unique sequence derived from the *prrF1-prrF2* intergenic region (Oglesby-Sherrouse and Vasil, 2010; Reinhart et al., 2015), a recent study showed that PrrH is not required for acute murine lung infection (Reinhart et al., 2017). Notably, the region unique to PrrH is highly conserved across *P. aeruginosa* strains of clinical origin, suggesting that a role for PrrH could be found using alternative infection models (Reinhart et al., 2017). The finding of heme-regulated sRNAs in both *L. monocytogenes* and *P. aeruginosa* raises the possibility of a more widespread role for sRNA-mediated control in the response of bacterial pathogens to heme.

## Author Contributions

PdS, PM-G, DS, and BK: conceived and designed the experiments. PdS, PM-G, DS, J-HC, MB, and EL: performed the experiments. PdS, PM-G, DS, J-HC, MB, EL, and BK: analyzed the data. PdS and BK: wrote the paper. All authors read and approved the final manuscript.

## Conflict of Interest Statement

The authors declare that the research was conducted in the absence of any commercial or financial relationships that could be construed as a potential conflict of interest.
