# Supplementary material for: The Small Regulatory RNAs LhrC1–5 Contribute to the Response of Listeria monocytogenes to Heme Toxicity
Source: Front Microbiol. 2018 Mar 27;9:599. doi: 10.3389/fmicb.2018.00599 (PMC5880928; doi:10.3389/fmicb.2018.00599)
Supplement: Supplementary file 1 [file Data_Sheet_1.DOCX]

Supplementary Material

The Small Regulatory RNAs LhrC1-5 Contribute to the Response of *Listeria monocytogenes* to Heme Toxicity

Patrícia Teixeira dos Santos^1^, Pilar Menendez-Gil^1^, Dharmesh Sabharwal^1^, Jens-Henrik Christensen^1^, Maja Zacho Brunhede^1^, Eva Maria Sternkopf Lillebæk^1^, Birgitte Haahr Kallipolitis^1*^

^1^ Department of Biochemistry and Molecular Biology, University of Southern Denmark, Odense M, Denmark

*** Correspondence:**Birgitte H. Kallipolitis
bhk@bmb.sdu.dk

**
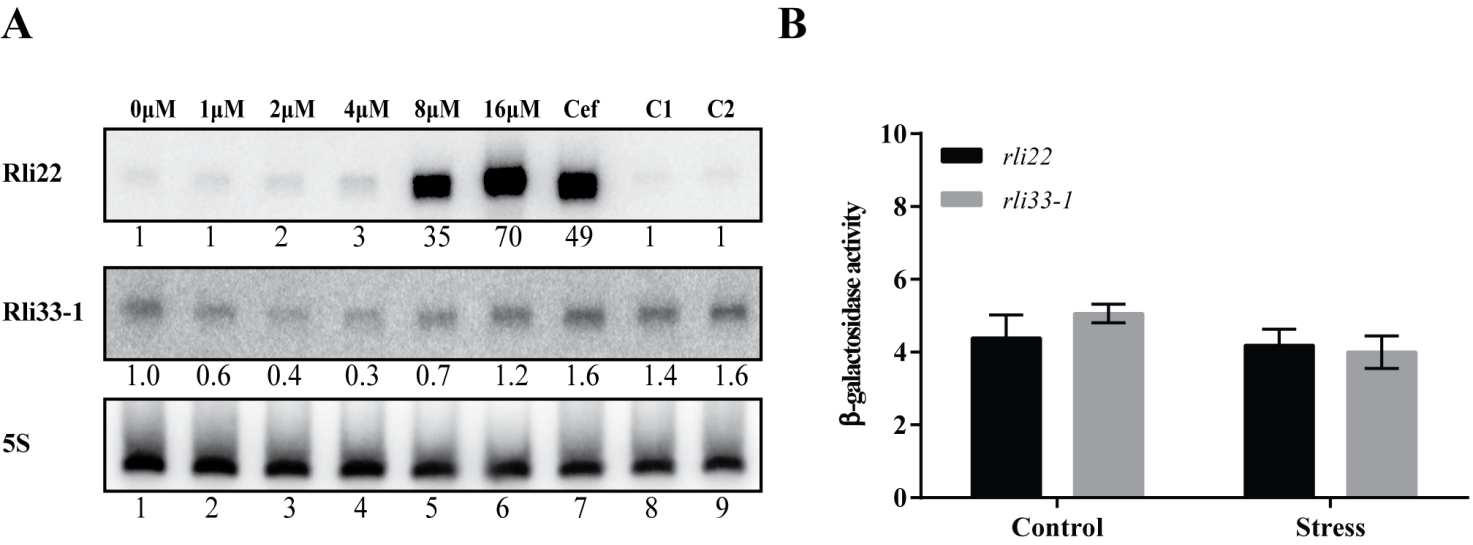
**

**Supplementary Figure S1:** Testing the expression of Rli22 and Rli33-1 during hemin stress. (**A**) Northern blot analysis of Rli22 and Rli33-1 expression. Samples were taken from *L. monocytogenes* LO28 wild-type cultures stressed with increasing concentrations of hemin (lanes 1 to 6), with a sub-inhibitory concentration of cefuroxime (4 μg/mL) (lane 7) or with the hemin dissolvent NaOH (the same volume used to dissolve 8 and 16 μM hemin – lanes 8 and 9). Northern blot was probed for Rli22, Rli33-1 and 5S rRNA as a loading control. Relative levels of Rli22 and Rli33-1 (normalized to 5S) are shown below each lane. (**B**) Transcriptional reporter gene fusions of *rli22* and *rli33-1* promoters. Plasmids containing promoter regions of *rli22* or *rli33-1* fused to *lacZ* (Mollerup et al., 2016) were transformed into LO28 wild-type. The resulting strains were grown up to OD_600_ = 0.35 and stressed with hemin (8 μM) after control samples had been taken (Control). Further samples for a following β-galactosidase assay were withdrawn after 2 hours (Stress). Results are the average of three biological replicates, each carried out in technical duplicates.

**
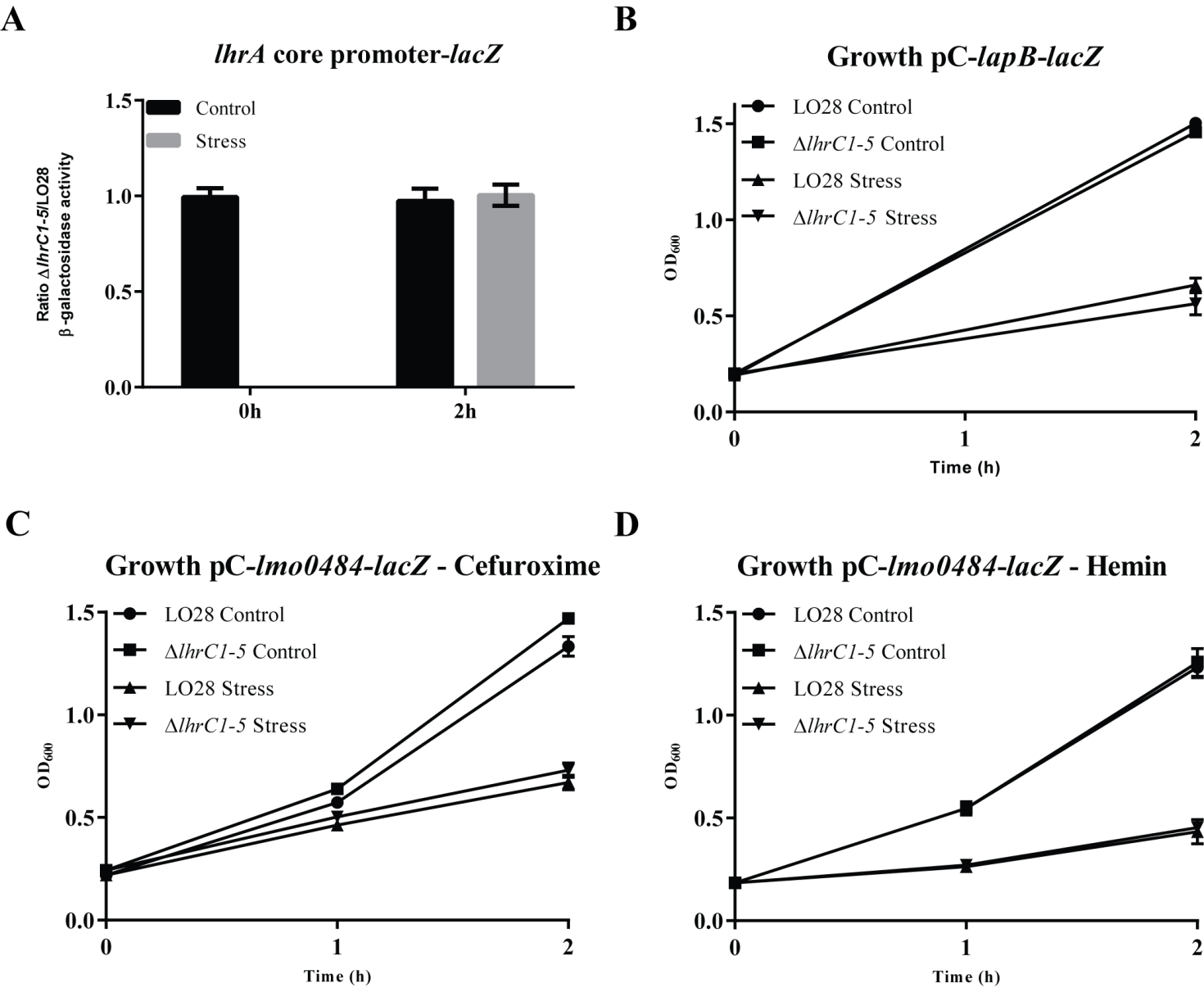
**

**Supplementary Figure S2:** (**A**) The *lhrA* core promoter is not affected by LhrC1-5 upon hemin exposure. In a previous study, the *lhrA* core promoter was fused to *lacZ* in the transcriptional fusion vector pTCV-lac (Sievers et al., 2014). β-galactosidase activity of LO28 wild-type and Δ*lhrC1-5* containing this plasmid was similar under control conditions as well as after 2 hours of hemin stress. Results are the average of three biological replicates, each in technical duplicates. (**B-D**) The growth of wild-type and Δ*lhrC1-5* cultures assessed in the β-galactosidase assay shown in (**B**) Figure 3B, (**C**) Figure 7A or (**D**) Figure 8A. Growth of the wild-type and Δ*lhrC1-5* strain was comparable under each of the conditions tested (i.e. control, cefuroxime or hemin stress, respectively).

**
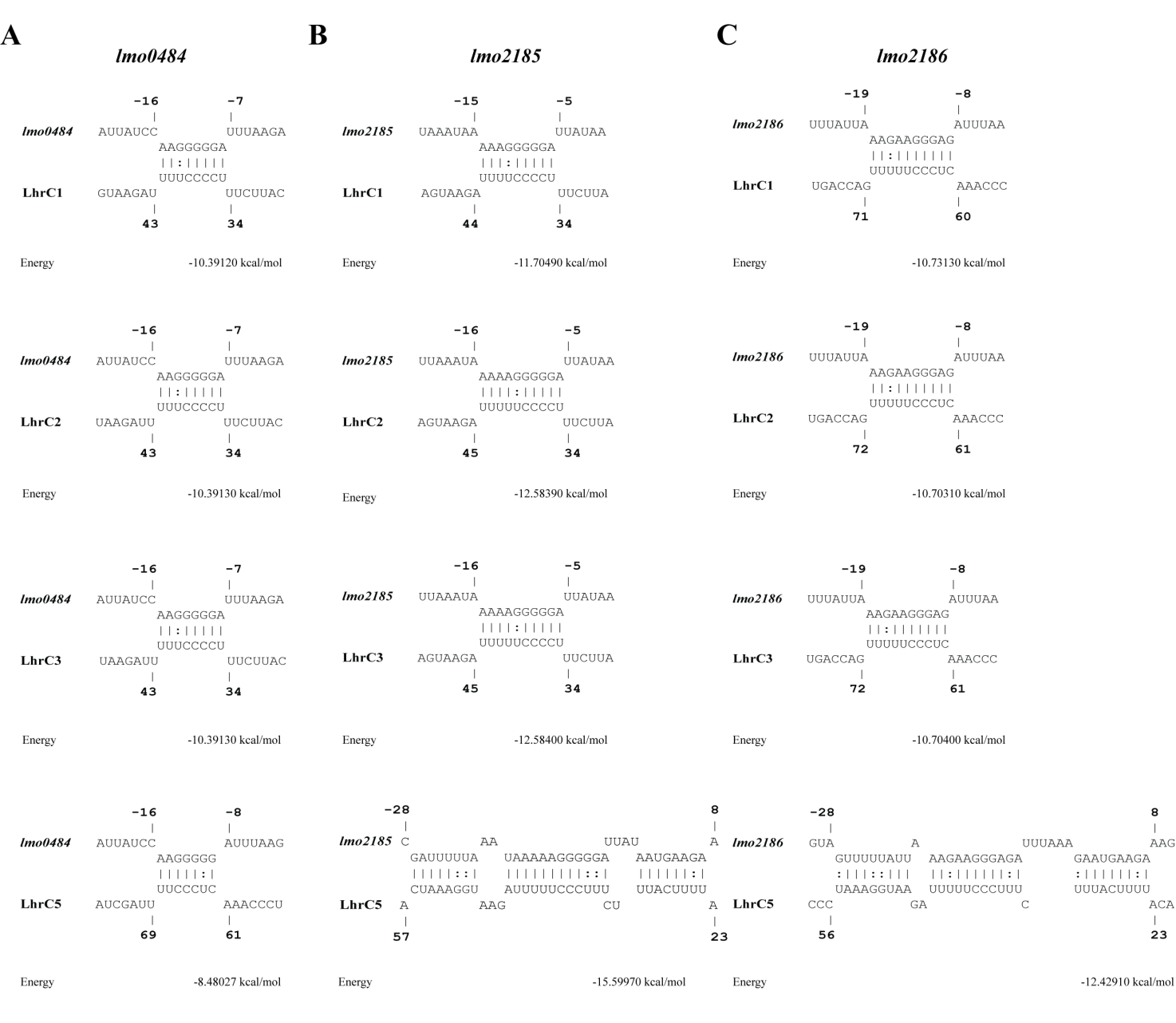
**

**Supplementary Figure S3:** *In silico* predictions of LhrC1, LhrC2, LhrC3 and LhrC5 interactions with mRNAs encoded from (**A**) *lmo0484*, (**B**) *lmo2185* or (**C**) *lmo2186*. According to the IntaRNA Software (Busch et al., 2008; Wright et al., 2014; Mann et al., 2017), the CU-rich regions of the LhrCs are predicted to bind to the sequence spanning the SD regions of the target mRNAs. The nucleotides of *lmo0484*, *lmo2185* and *lmo2186* are numbered relative to the translation start site, and the nucleotides of LhrC1, LhrC2, LhrC3 and LhrC5 are numbered relative to the 5´-end of the sRNA.

**
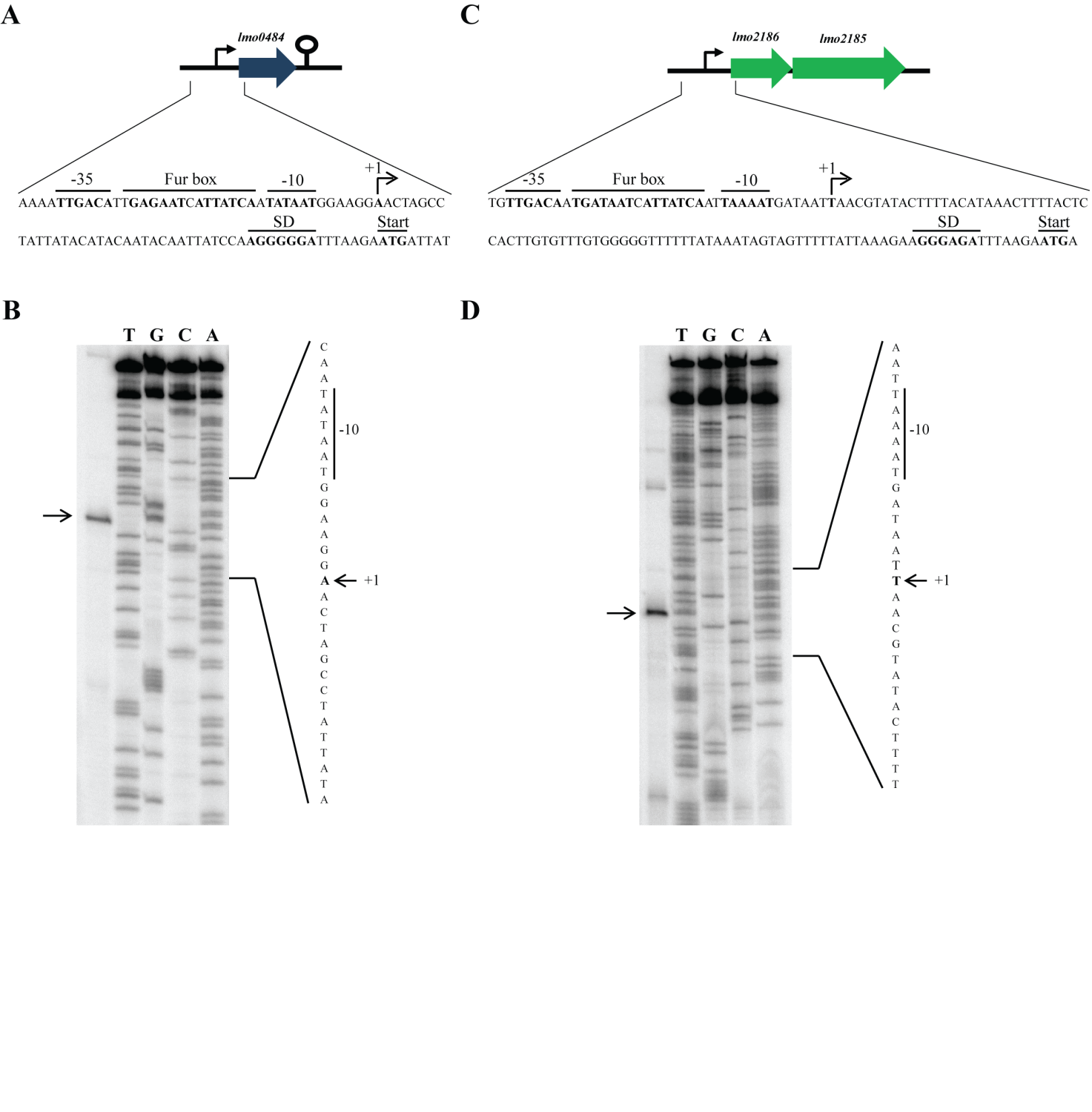
**

**Supplementary Figure S4:** Mapping the transcriptional start site of *lmo0484* and *lmo2186*-*lmo2185*. (**A**) and (**C**) Illustration of the genomic location and promoter region sequence for (**A**) *lmo0484* and (**C**) *lmo2186*-*lmo2185*. The *lmo0484* gene is transcribed monocistronically (Toledo-Arana et al., 2009), whereas the *lmo2186*-*lmo2185* genes are the first genes of the heme acquisition operon and are thus co-transcribed (Toledo-Arana et al., 2009; McLaughlin et al., 2012). Transcriptional start sites (+1) are marked by an arrow. The -35 box, Fur box, -10 box, the predicted SD sequence and the start codon are marked in bold. The lollipop structure denotes a transcription terminator structure. (**B**) and (**D**) Primer extension analysis to determine the 5´-end of (**B**) *lmo0484* and (**D**) *lmo2186*-*lmo2185* mRNAs. Total RNA from Δ*lhrC1-5* grown to OD_600_ = 0.35 and subjected to cefuroxime stress (4 μg/ml) for 1 hour was used for the experiment. T, G, C and A lanes represent the sequencing ladders. The transcriptional start site (+1) is marked in bold and the -10 box is indicated.

**
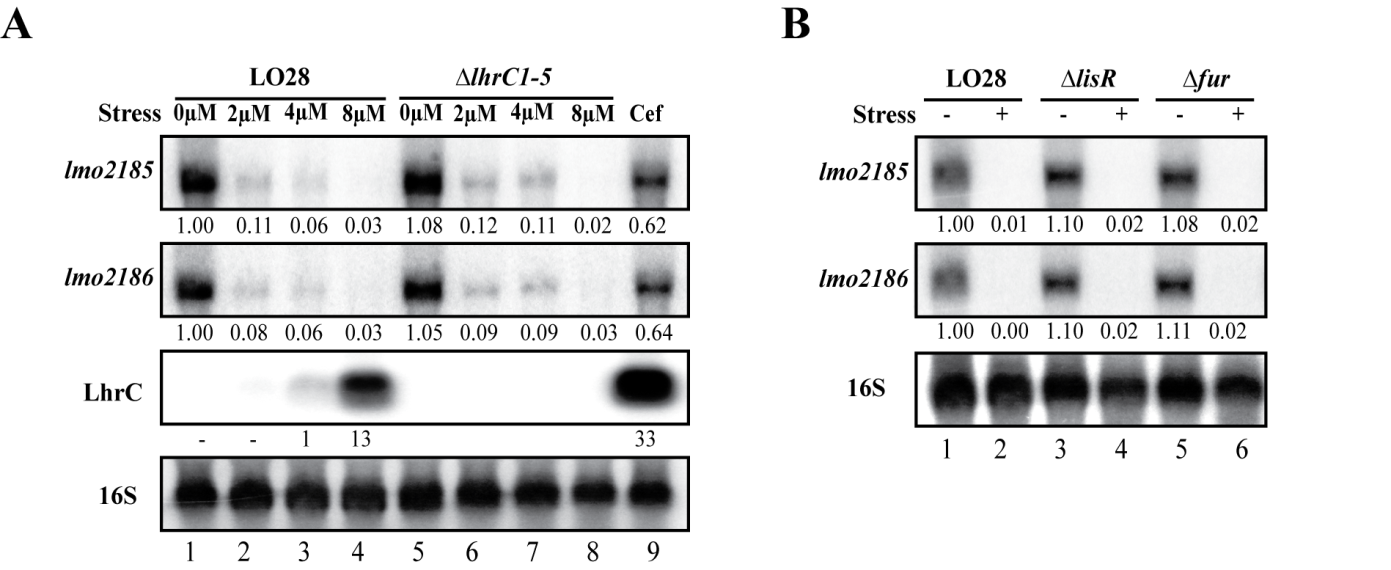
**

**Supplementary Figure S5:** Assessing the effect of LhrC1-5 on *lmo2186*-*lmo2185* mRNA under hemin stress. (**A**) Northern blot analysis of *lmo2186*-*lmo2185* mRNA after exposure to various concentrations of hemin. Samples were taken from LO28 wild-type and Δ*lhrC1-5* cultures exposed to 0, 2, 4 or 8 μM hemin stress for 1 hour. LO28 wild-type exposed to 4 μg/mL cefuroxime was used as a control. Northern blots were probed for *lmo2185* and *lmo2186* mRNA, LhrC1-5 and 16S rRNA (loading control). Relative levels of *lmo2185* mRNA, *lmo2186* mRNA, and LhrC1-5 (normalized to 16S) are shown below each lane. (**B**) Testing the role of LisR and Fur in the heme-dependent repression of *lmo2185* and *lmo2186*. Samples were taken from LO28 wild-type, Δ*lisR* and Δ*fur* cultures exposed for 1 hour to 8 μM hemin stress (+) as well as from non-stressed cultures (-). Northern blots were probed for *lmo2185* and *lmo2186* mRNA, and 16S rRNA (loading control). Relative levels of *lmo2185* and *lmo2186* mRNA normalized to 16S are shown below each lane.

**Supplementary Table S1:** Primers used in this study

| Name | Sequence (5’→ 3’) | Further information |
| --- | --- | --- |
| Cloning for in-frame deletion | | |
| 0484-1 | GGGGGAATTCAGTCTGGATTCCGGTGGTTG | Forward primer for upstream flanking region of *lmo0484*. EcoRI restriction enzyme site. |
| 0484-2 | CAATAATCATTCTTAAATCCCCCTTG | Reverse primer for upstream flanking region of *lmo0484*. |
| 0484-3 | GGATTTAAGAATGATTATTGGTTCATGTGCAAAACCCTGTG | Forward primer for downstream flanking region of *lmo0484*. Anneals with 0484-2. |
| 0484-4 | CCCCGGATCCGTTTAATCCCCCCTTAAATGATAAAC | Reverse primer for downstream flanking region of *lmo0484*. BamHI restriction enzyme site. |
| 0484-5 | CTTCAAATCAGTCGGCCCAC | Forward primer for verification of chromosomal deletion of *lmo0484*. |
| 0484-6 | CACGGCTCTACTTTGCTGTG | Reverse primer for verification of chromosomal deletion of *lmo0484*. |
| fur-1 | GGGGAAGCTTGTAGACAGCCTATTGCTTTTGTG | Forward primer for upstream flanking region of *fur*. HindIII restriction enzyme site. |
| fur-2 | GTTGTGCTTTAATGCGTCCAATAC | Reverse primer for upstream flanking region of *fur*. |
| fur-3 | TGGACGCATTAAAGCACAACGATCATCGTCTGACTTTCCAAG | Forward primer for downstream flanking region of *fur*. Anneals with fur-2. |
| fur-4 | CCCCGGATCCCGTTTCCTCCACAAATCCTATTC | Reverse primer for downstream flanking region of *fur*. BamHI restriction enzyme site. |
| fur-5 | GTTGTGGCAATTATCGGTGC | Forward primer for verification of chromosomal deletion of *fur*. |
| fur-6 | GTATTCGCGGATAAACCTCTC | Reverse primer for verification of chromosomal deletion of *fur*. |
| pAUL-1 | ATGATTACCGCCCAAGCTTG | Forward primer for verification of plasmid construct |
| pAUL-2 | CAGGACGTTGTAAAACGACG | Reverse primer for verification of plasmid construct |
| *lacZ* fusions | | |
| 0484-F-translational-2 | GGGGGAATTCCTTGCTTTTTTCAAGAACAATAGTAAAATAAGTTAAACTAGCCTATTATACATACAATAC | Forward primer, translational fusion of LhrA core promoter + *lmo0484* (-48 to +47, relative to translation start site) in pCK-lac. EcoRI restriction enzyme site. |
| BamHI-R-lmo0484 | CCCCGGATCCTCTGCTGCGCCTTTTTCTAC | Reverse primer, translational fusion of LhrA core promoter + *lmo0484* (-48 to +47, relative to translation start site) in pCK-lac. BamHI restriction enzyme site. |
| Vlac-1 | GTTGAATAACACTTATTCCTATC | Flanking forward primer to check insertions in pCK-lac. |
| -40 primer | GTTTTCCCAGTCACGACGTTGTAAAACGACGG | Flanking reverse primer to check insertions in pCK-lac. |
| Primer extensions | | |
| EcoRI-F-lmo0484 | GGGGGAATTCCCAAACCCATTCCCTAAAATTG | Forward primer, *lmo0484* sequencing ladder. |
| lmo0484-PE | CTGACGGATAACGTGCTCTG | Reverse primer, *lmo0484* sequencing ladder. |
| EcoRI-F-lmo2186 | GGGGGAATTCGATCGCTTTTCTCAAATAGAAGAG | Forward primer, *lmo2186* sequencing ladder. |
| BamHI-R-lmo2186-2 | CCCCGGATCCGCAGCAAAAACTAAAACTTTCTTC | Reverse primer, *lmo2186* sequencing ladder. |
| NB probes | | |
| LhrC_probe_specific | AATGAAAATGTTTTGCTTGTTGTTAGCTTAT | Single stranded probe for LhrC |
| Rli22 NB probe new | GTAAAACTAATTGTATTCCTTGCTTAC | Single stranded probe for Rli22 |
| Rli33-1 NB probe | GTAATGGATTGTCATTGCTTATG | Single stranded probe for Rli33-1 |
| *tcsA* ss NB probe | GTAAGCCTTCCCATGCTGATTGGTTAAACGAACGGTC | Single stranded probe for *tcsA* mRNA |
| *oppA* ss NB probe | GGAATGCGAATAGTGAGTTAATGTAAGCAGTTGG | Single stranded probe for *oppA* mRNA |
| *lmo0484* NB probe | CTTTTTCTACCTTAATCGTATTAGTTAC | Single stranded probe for *lmo0484* mRNA |
| *lmo2185* NB probe | GTTGTACCTGGATCAGGCTTTG | Single stranded probe for *lmo2185* mRNA |
| *lmo2186* NB probe | GTTAATCCAGTAGAAAGAAACGAAAAAC | Single stranded probe for *lmo2186* mRNA |
| 5S rRNA | GAGAAGCTTAACTACCGTGTTCGGGATGGGAACGG | Single stranded probe for 5S rRNA |
| 16S rRNA | GGCCATTACCCTACCAACTAGCTAATGCAC | Single stranded probe for 16S rRNA |
| RT-qPCR | | |
| 0484-F-qPCR-2 | CACACGGCAGAACAAAAGACAC | Forward primer for *lmo0484*. |
| 0484-R-qPCR-2 | GATTGCATTTCCTACGATGCCTTTG | Reverse primer for *lmo0484*. |
| rpoB_fw | CGTCGTCTTCGTTCTGTTGG | Forward primer for the reference gene *rpoB*. |
| rpoB_rev | GTTCACGAACCACACGTTCC | Reverse primer for the reference gene *rpoB*. |
| tpi_fw | AACACGGCATGACACCAATC | Forward primer for the reference gene *tpi*. |
| tpi_rev | CACGGATTTGACCACGTACC | Reverse primer for the reference gene *tpi*. |
| EMSAs and structural probing | | |
| T7-0484-F-in vitro | GGGGTAATACGACTCACTATAGGGAACTAGCCTATTATACATACAATAC | Forward primer, synthesis of *lmo0484* DNA with T7 promoter to be transcribed into RNA. |
| 0484-R-in vitro | TCTGCTGCGCCTTTTTCTAC | Reverse primer, synthesis of *lmo0484* DNA with T7 promoter to be transcribed into RNA. |
| T7-2186-F-in vitro | GGGGTAATACGACTCACTATAGGGTAACGTATACTTTTACATAAACTTTTAC | Forward primer, synthesis of *lmo2186* DNA with T7 promoter to be transcribed into RNA. |
| 2186-R-in vitro | GCAGCAAAAACTAAAACTTTCTTC | Reverse primer, synthesis of *lmo2186* DNA with T7 promoter to be transcribed into RNA. |
| T7-2185-F-in vitro | GGGGTAATACGACTCACTATAGGGCTGGGGCAGGGTTAATTTTAC | Forward primer, synthesis of *lmo2185* DNA with T7 promoter to be transcribed into RNA. |
| 2185-R-in vitro | AGTGTCAAAGCTAAGAAAGCTAC | Reverse primer, synthesis of *lmo2185* DNA with T7 promoter to be transcribed into RNA. |
| Fw T7 LhrC4 in vitro | GGGGGAATTCTAATACGACTCACTATAGGGATAAGCTAACAACAAACAAAACATTTTCATTCTTCTCCCCCCTTTTAGAATGAAAATCCC | Forward primer, synthesis of *lhrC4* DNA with T7 promoter to be transcribed into RNA. Use in combination with Rev LhrC4 in vitro. |
| Rev LhrC4 in vitro | GGGGGGATCCAAAAAAACCGATGCGGAAAAGGGAGTAAACCGCATCGGTCAAAAAAGGGAGTTTGGGATTTTCATTCTAAAAGGGG | Reverse primer, synthesis of *lhrC4* DNA with T7 promoter to be transcribed into RNA. Use in combination with Fw T7 LhrC4 in vitro. |
| lhrC4_mut_2_fw | GGGGGAATTCTAATACGACTCACTATAGGGATAAGCTAACAACAAACAAAACATTTTCATTCTTCTCCCCCCTTTTAGAATGAAAATAGAACAG | Forward primer for making LhrC_mut_2. Use in combination with lhrC4_mut_2_rev. |
| lhrC4_mut_2_rev | GGGGGGATCCAAAAAAACCGATGCGGAAAAGGGAGTAAACCGCATCGGTCGGGGTTCCTTCTGTTCTATTTTCATTCTAAAAG | Reverse primer for making LhrC_mut_2. Use in combination with lhrC4_mut_2_fw. |
| lhrC4_mut_3_fw | GGGGGAATTCTAATACGACTCACTATAGGGATAAGCTAACAACAAACAAAACATTTTCATTCTTCTCCCCCCTTTTAGAATGAAAATCCCAAAC | Forward primer for making LhrC_mut_3. Use in combination with lhrC4_mut_3_rev. |
| lhrC4_mut_3_rev | GGGGGGATCCAAAAAAACCGATGCGGTTTTCCCTCTAGTCCGCATCGGTCAAAAAAGGGAGTTTGGGATTTTCATTCTAAAAG | Reverse primer for making LhrC_mut_3. Use in combination with lhrC4_mut_3_fw. |
| lhrC4_mut_4_fw | GGGGGAATTCTAATACGACTCACTATAGGGATAAGCTAACAACAAACAAAACATTTTCATTCTAATGGCGGGAAAAAGAATGAAAATCCCAAAC | Forward primer for making LhrC_mut_4. Use in combination with lhrC4_mut_4_rev. |
| lhrC4_mut_4_rev | GGGGGGATCCAAAAAAACCGATGCGGAAAAGGGAGTAAACCGCATCGGTCAAAAAAGGGAGTTTGGGATTTTCATTCTTTTTC | Reverse primer for making LhrC_mut_4. Use in combination with lhrC4_mut_4_fw. |
| lmo0484_MUT_fw | GGGGTAATACGACTCACTATAGGGAACTAGCCTATTATACATACAATACAATTATCCAACGGGCATTTAAGAATGATTA | Forward primer for making lmo0484_MUT. Use in combination with lmo0484_MUT_rev. |
| lmo0484_MUT_rev | TCTGCTGCGCCTTTTTCTACCTTAATCGTATTAGTTACAATAATCATTCTTAAATGCCCG | Reverse primer for making lmo0484_MUT. Use in combination with lmo0484_MUT_fw. |
| lhrC4_loopA_MUT_fw | ggggTAATACGACTCACTATAGGGATAAGCTAACAACAAACAAAACATTTTCATTCTTCTCGCCCGTTTTAGAATGAAAATCCC | Forward primer for making lhrC4_loopA_MUT. Use in combination with lhrC4_loopA_MUT_rev. |
| lhrC4_loopA_MUT_rev | CCCCAAAAAAACCGATGCGGAAAAGGGAGTAAACCGCATCGGTCAAAAAAGGGAGTTTGGGATTTTCATTCTAAAACGGGC | Reverse primer for making lhrC4_loopA_MUT. Use in combination with lhrC4_loopA_MUT_fw. |

Supplementary Table S2: Strains used in this study.

| Strain | Origin |
| --- | --- |
| *Listeria monocytogenes* serotype 1/2c strain LO28 | (Vazquez-Boland et al., 1992) |
| LO28Δ*lhrC1-5* | (Sievers et al., 2014) |
| LO28Δ*lisR* | (Kallipolitis et al., 2003) |
| LO28Δ*lmo0484* | This study |
| LO28Δ*fur* | This study |
| *E. coli* TOP10 | Invitrogen |

**References:**

Busch, A., Richter, A.S., and Backofen, R. (2008). IntaRNA: efficient prediction of bacterial sRNA targets incorporating target site accessibility and seed regions. *Bioinformatics* 24(24)**,** 2849-2856. doi: 10.1093/bioinformatics/btn544.

Kallipolitis, B.H., Ingmer, H., Gahan, C.G., Hill, C., and Sogaard-Andersen, L. (2003). CesRK, a two-component signal transduction system in Listeria monocytogenes, responds to the presence of cell wall-acting antibiotics and affects beta-lactam resistance. *Antimicrob Agents Chemother* 47(11)**,** 3421-3429.

Mann, M., Wright, P.R., and Backofen, R. (2017). IntaRNA 2.0: enhanced and customizable prediction of RNA-RNA interactions. *Nucleic Acids Res*. doi: 10.1093/nar/gkx279.

McLaughlin, H.P., Xiao, Q., Rea, R.B., Pi, H., Casey, P.G., Darby, T., et al. (2012). A putative P-type ATPase required for virulence and resistance to haem toxicity in Listeria monocytogenes. *PLoS One* 7(2)**,** e30928. doi: 10.1371/journal.pone.0030928.

Mollerup, M.S., Ross, J.A., Helfer, A.C., Meistrup, K., Romby, P., and Kallipolitis, B.H. (2016). Two novel members of the LhrC family of small RNAs in Listeria monocytogenes with overlapping regulatory functions but distinctive expression profiles. *RNA Biol* 13(9)**,** 895-915. doi: 10.1080/15476286.2016.1208332.

Sievers, S., Sternkopf Lillebaek, E.M., Jacobsen, K., Lund, A., Mollerup, M.S., Nielsen, P.K., et al. (2014). A multicopy sRNA of Listeria monocytogenes regulates expression of the virulence adhesin LapB. *Nucleic Acids Res* 42(14)**,** 9383-9398. doi: 10.1093/nar/gku630.

Toledo-Arana, A., Dussurget, O., Nikitas, G., Sesto, N., Guet-Revillet, H., Balestrino, D., et al. (2009). The Listeria transcriptional landscape from saprophytism to virulence. *Nature* 459(7249)**,** 950-956. doi: 10.1038/nature08080.

Vazquez-Boland, J.A., Kocks, C., Dramsi, S., Ohayon, H., Geoffroy, C., Mengaud, J., et al. (1992). Nucleotide sequence of the lecithinase operon of Listeria monocytogenes and possible role of lecithinase in cell-to-cell spread. *Infect Immun* 60(1)**,** 219-230.

Wright, P.R., Georg, J., Mann, M., Sorescu, D.A., Richter, A.S., Lott, S., et al. (2014). CopraRNA and IntaRNA: predicting small RNA targets, networks and interaction domains. *Nucleic Acids Res* 42(Web Server issue)**,** W119-123. doi: 10.1093/nar/gku359.
